# Diurnal variation in neurovascular coupling and the impact of sleep quality: a UK Biobank study

**DOI:** 10.1093/sleep/zsaf256

**Published:** 2025-08-29

**Authors:** Sheng Yang, Alastair John Stewart Webb

**Affiliations:** Wolfson Centre for Prevention of Stroke and Dementia, Nuffield Department of Clinical Neurosciences, University of Oxford, John Radcliffe Hospital, Headley Way, Oxford OX39DU, United Kingdom; Wolfson Centre for Prevention of Stroke and Dementia, Nuffield Department of Clinical Neurosciences, University of Oxford, John Radcliffe Hospital, Headley Way, Oxford OX39DU, United Kingdom; Imperial College London, Department of Brain Sciences, London, United Kingdom

**Keywords:** neurovascular coupling, sleep quality, circadian rhythms, neuroimaging, cerebrovascular risk

## Abstract

**Study Objectives:**

Cerebrovascular events are more frequent in the morning, coinciding with disturbed circadian rhythms and poor sleep quality. Neurovascular coupling (NVC), a marker of neuronal activity and endothelium-dependent cerebrovascular function, may mediate the relationship between cerebrovascular dysfunction and chronic cerebrovascular disease. This study aims to determine whether NVC shows circadian variation and whether it is associated with sleep quality.

**Methods:**

Functional MRI scans from the UK Biobank were adapted to assess NVC at different time points throughout the day. Analysis of Variance (ANOVA) with Tukey post hoc tests examined NVC differences between 3 and 2-h time blocks starting at 8 am Cosinor models assessed rhythmicity in NVC. General linear models evaluated the impact of sleep quality (a composite score and individual markers: snoring, insomnia, duration, chronotype, and daytime sleepiness) on NVC, adjusting for age, sex, and vascular risk factors.

**Results:**

Among 36 801 participants, NVC was significantly lower between 11 am and 2 pm, with a consistent pattern across ages in men but a midday decline in younger women (<60 years). While the composite measure of sleep quality risk was not associated with a change in NVC (*p* = .12), a diagnosis of insomnia (*p* = .04) or sleep apnea (*p* = .03) was associated with lower NVC.

**Conclusions:**

The observed reduction in NVC in the late morning and its association with objective measures of impaired sleep quality suggest a potential role for endothelial dysfunction, potentially contributing to the associated increased cerebrovascular risk.

**Statement of Significance**

Cerebrovascular fluctuations with time-of-day and with sleep quality could offer insights into stroke mechanisms. This study provides the first large-scale evidence that neurovascular coupling (NVC), a functional marker of cerebrovascular health, follows a diurnal pattern characterized by a reproducible midday dip independent of age, sex, and vascular risks. This suggests a period of potential vulnerability in the cerebral blood supply. Furthermore, a history of stroke and sleep disorders such as sleep apnea are associated with impaired NVC, indicating a disruption of the brain’s natural rhythms. These findings highlight translational opportunities to optimize sleep and align preventive strategies with diurnal physiology to reduce cerebrovascular risk. Remaining knowledge gaps include the need for 24-h assessments and longitudinal studies to clarify causal pathways.

## Graphical Abstract

**Graphical Abstract:**
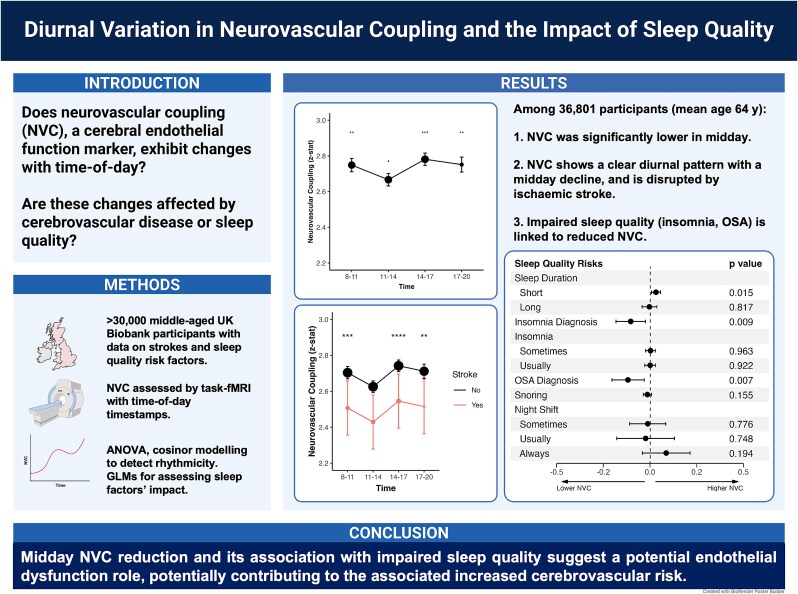

## Introduction

The incidence of stroke is highest in the morning [1, 2], while individuals with sleep disruptions, whether insufficient sleep duration or poor sleep quality, are at an elevated risk for cardiovascular events and dementia [3–11]. Diurnal variation in cerebrovascular function may underlie the impact of the 24-h cycle on health and productivity, through reduced cerebrovascular resilience to cardiovascular stress at different times of day and reduced blood flow responses to neuronal demand [12]. Despite the ubiquity of biological rhythms in cardiovascular function [13, 14], from the morning peak in sympathomimetic hormones to a nocturnal fall in body temperature [14, 15], there is limited evidence regarding diurnal patterns and endogenous circadian rhythms in cerebrovascular function [12].

One commonly used marker of cerebrovascular endothelial function is cerebrovascular reactivity (CVR) [16], which measures the cerebral blood flow (CBF) response to vascular stimuli, such as a carbon dioxide challenge [17–20]. Although evidence exists for reduced CBF velocity at midday and reduced CVR and autoregulation in the morning, these studies were small and in specific patient groups [12, 18, 21, 22], while measurement of CVR in large, population-based studies remains challenging [16].

Neurovascular coupling (NVC), which assesses the brain's blood flow response to neural activity, is easier to measure, and is dependent upon both cerebrovascular endothelial function and neuronal activity [20, 23]. Disruptions in NVC are associated with cardiovascular risk factors, such as hypertension, diabetes [24], and cerebrovascular disease, including ischemic stroke, cerebral small vessel disease (SVD) [17, 25, 26], Alzheimer’s disease [17, 27], and vascular dementia [28]. While some small studies suggested diurnal variation in NVC, the evidence is limited and inconsistent [12, 29–31].

This study tested the hypothesis that NVC, as a marker of cerebrovascular compensatory mechanisms, would be reduced in association with diurnal factors known to be associated with increased cerebrovascular risk. For example, that NVC would show a circadian pattern with a morning decline; that individuals with a history of ischemic stroke or greater burden of SVD would exhibit disrupted NVC rhythmicity; that impaired sleep quality would be associated with diminished NVC or disrupted NVC rhythmicity.

## Materials and Methods

### Participants selection

UK Biobank (UKB) is a large, community-based prospective cohort study comprising demographic, lifestyle, clinical records, and imaging data from over 500 000 middle-aged participants recruited from 22 centers across the United Kingdom [32].

This cross-sectional analysis includes participants with available NVC data (UKB ID: 25042). The NVC measure used in this study corresponds to the median *z*-statistic of the task-evoked functional Blood Oxygen Level Dependent MRI (BOLD) response within a group-averaged mask, derived from the contrast between simple images versus baseline fixation, as described previously [24]. The resulting BOLD signal was pre-processed, modeled, and analyzed to derive NVC (detailed in Table S1). This *z*-statistic reflects both the amplitude and consistency of the BOLD signal change and served as a validated proxy for NVC. This methodology has shown associations between reduced NVC, vascular risk factors, vascular dementia, and SVD [17, 26, 33].

Participants were excluded if they had conditions potentially confounding the analysis, such as central nervous system infection or inflammation, brain tumors, or lupus (identified through International Classification of Diseases-10 codes), or if they exhibited excessive head motion during fMRI (mean head motion >1 mm, UKB ID: 25742). Additional exclusions included extreme systolic blood pressure (SBP < 80 mmHg or >220 mmHg) or diastolic blood pressure (DBP < 40 mmHg or >120 mmHg) and cases where reported SBP ≤ DBP.

### Neuroimaging data

The following MRI-related data were extracted from each participant as a single time-point measurement, including: NVC values (median *z*-statistic), scan start time (time-of-day), and total white matter hyperintensities normalized by white matter volume (nWMH). The time-of-day was rounded to the second decimal place with stratifications into 3 and 2-h intervals for analysis [34]. Volume of nWMH was divided into quartiles to represent SVD burden, with quartiles representing increasing SVD severity [35]. Details of the data is summarized in Table S2.

### Demographic data

Age at the time of scan, sex, ethnicity, health conditions, and sleep quality risk factors were included in this analysis. Vascular health was assessed through SBP, DBP, waist–hip ratio, and disease history identified through ICD-10 codes, combined with self-reported history of diabetes and ischemic stroke (Table S2).

In this analysis, sleep quality risk factors included self-reported snoring, insomnia, sleep duration, sleep chronotype (morning or evening preference), daytime sleepiness, and frequency of night shifts. Diagnoses of obstructive sleep apnea (OSA) or insomnia were identified with ICD-10 codes. Sleep duration was classified into short (<7 h), optimal (7–8 h), and long (≥9 h) [36]. A composite Sleep Quality Risk Score, ranging from 0 to 5, was calculated based on five sleep-related factors: snoring, insomnia symptoms, sleep duration, sleep chronotype (preference for mornings or evenings), and daytime sleepiness [37], with higher scores reflecting healthier sleep patterns. Based on this score, participants were grouped into three sleep pattern groups: healthy (≥4), intermediate (2–3), and poor (≤1).

### Statistical analysis

Baseline demographics of the included subjects were presented as mean ± standard deviation for continuous variables and counts with percentage for categorical variables. Quantile–Quantile plots assessed continuous data normality; non-normal variables were log-transformed before analysis.

Circadian patterns in NVC were assessed using ANOVA and cosinor analysis, two established methods for detecting rhythmicity in biological time-series data [38–43]. One-way ANOVA with Tukey's honestly significant difference (HSD) multiple pairwise comparisons tests identified group differences in NVC across 2 and 3-h time bins (*α* = 0.05). The effects of age, sex, cerebrovascular diseases (ischemic stroke and burden of SVD), and sleep quality risk factors on NVC were assessed in subgroup ANOVA [39]. Cosinor analysis modeled time-of-day as a continuous variable, assuming 12 and 24-h periodicity to reflect the diurnal and ultradian rhythms observed in human circadian physiology [39, 43]. NVC data were modeled using a single-component cosinor wave, fitted using least squares regression [44]. The goodness-of-fit of the cosinor models was compared against linear regression using Akaike information criteria. From the cosinor analysis, the following oscillation parameters were derived: midline estimating statistic of rhythm (MESOR, the mean value where the oscillations occur, equivalent to an equilibrium point), amplitude (the distance between the oscillation peak and MESOR, reflecting the magnitude of rhythmic variation), and acrophase (the timing of the peak). Rhythmicity was statistically validated with *F*-tests, where the null hypothesis (*H*_0_) posited the absence of rhythm (zero-amplitude) [39, 42–44].

General linear models (GLMs) evaluated the impacts of sleep quality risk factors (both individual and composite) on overall (unstratified) NVC, controlling for age, sex, and cardiovascular risk factors. Results were reported as standardized coefficients (*β*) with corresponding *p*-values, adjusted for false discovery rate (two-tailed tests, *α* = 0.05).

All analysis was performed in R with the following packages: *Cosinor2* (rhythmicity differentiation in cosinor models), *GLMMcosinor* (cosinor modeling and parameters estimation), *ggeffects*, and *forester* (plotting) [44–48].

## Results

### Study selection

After excluding participants with missing NVC or time-of-day data, confounding diagnoses, extreme blood pressure values, or excessive head motion, 36 801 subjects were included in this cross-sectional analysis (Figure S1). Due to non-normal distributions, nWMH volume was log-transformed (Figure S2). All included scans were conducted between 8:00 am and 8:00 pm. The mean age of participants was 63.99 ± 7.74 years, with an average time-of-day of scans of 13.51 ± 3.14 h (Table 1). Participants with a history of ischemic stroke or higher burden of SVD were older, had a greater percentage of males, and exhibited higher SBP, DBP, and WHR (Tables S3 and S4).

**Table 1:** Baseline demographic and clinical characteristics by time-of-day of MRI scan

**Variable**	**Total**	**Time-of-day intervals**				***P* value**
		**8:00–11:00**	**11:00–14:00**	**14:00–17:00**	**17:00–20:00**	
***N***	36 801	9762	10 300	10 414	6325	<.001
Age (years)	63.99 (7.74)	62.65 (7.81)	64.67 (7.72)	64.82 (7.54)	63.56 (7.67)	<.001
Male (*N*)	17 396 (47.3%)	5094 (52.2%)	4454 (43.2%)	4661 (44.8%)	3187 (50.4%)	<.001
White (*N*)	33 617 (91.3%)	8948 (91.7%)	9439 (91.6%)	9509 (91.3%)	5713 (90.3%)	<.01
SBP (mmHg)	139.16 (18.57)	137.92 (18.14)	138.39 (18.58)	140.59 (18.78)	139.97 (18.66)	<.001
DBP (mmHg)	78.00 (10.45)	78.16 (10.30)	77.09 (10.30)	78.38 (10.58)	78.61 (10.61)	<.001
Waist–hip ratio	0.88 (0.09)	0.88 (0.09)	0.87 (0.09)	0.88 (0.09)	0.88 (0.09)	<.001
Ischemic stroke history (*N*)	363 (1.0%)	90 (0.9%)	95 (0.9%)	115 (1.1%)	63 (1.0%)	.51
Normalized WMH volume^a^	0.01 (0.01)	0.01 (0.01)	0.01 (0.01)	0.01 (0.01)	0.01 (0.01)	<.001
Sleep quality risk score^b^	2.47 (0.82)	2.48 (0.83)	2.45 (0.80)	2.48 (0.81)	2.43 (0.83)	<.01
**Sleep duration (*N*)** ^**c**^						<.001
Normal	24 992 (68.5%)	6550 (67.4%)	7124 (69.6%)	6974 (67.8%)	4344 (69.5%)	
Short	8850 (24.3%)	2598 (26.7%)	2408 (23.5%)	2438 (23.7%)	1406 (22.5%)	
Long	2643 (7.2%)	576 (5.9%)	698 (6.8%)	867 (8.4%)	502 (8.0%)	
**Insomnia (*N*)** ^**d**^						<.001
Never/rarely	7963 (21.8%)	2237 (22.9%)	2082 (20.3%)	2154 (20.9%)	1490 (23.8%)	
Sometimes	16 831 (46.0%)	4378 (44.9%)	4756 (46.4%)	4892 (47.5%)	2805 (44.8%)	
Usually	11 756 (32.2%)	3129 (32.1%)	3403 (33.2%)	3263 (31.7%)	1961 (31.3%)	
Insomnia diagnosis (*N*)	618 (1.7%)	144 (1.5%)	144 (1.4%)	202 (1.9%)	128 (2.0%)	<.001
OSA diagnosis (*N*)	518 (1.4%)	115 (1.2%)	121 (1.2%)	175 (1.7%)	107 (1.7%)	<.001
Snorers (*N*)	12 278 (33.4%)	3457 (35.4%)	3303 (32.1%)	3423 (32.9%)	2095 (33.1%)	<.001
**Night shift work (*N*)** ^**e**^						0.22
Never/rarely	1117 (52.5%)	386 (53.5%)	245 (54.9%)	291 (51.7%)	195 (48.9%)	
Sometimes	574 (26.9%)	208 (28.8%)	107 (24.0%)	143 (25.4%)	116 (29.1%)	
Usually	169 (7.9%)	48 (6.7%)	41 (9.2%)	49 (8.7%)	31 (7.8%)	
Always	269 (12.6%)	79 (11.0%)	53 (11.9%)	80 (14.2%)	57 (14.3%)	

Continuous variables are shown as mean (SD) and categorical variables as count (%). SBP: systolic blood pressure; DBP: diastolic blood pressure; WMH: white matter hyperintensity; OSA: obstructive sleep apnea. Data were available in a: 98.5%, b: 90.2%, c: 99.1%, d: 99.3%, e: 5.8% of data, respectively.

### Age and sex differences in NVC

NVC was negatively associated with age (*β* = −0.07 [95% CI = −0.08 to −0.06] *p* < 10^−16^). Subjects younger than 60 years old had 1.51 per cent higher NVC compared to those aged 60–69 years, with a steeper decline of 9.1 per cent in participants over 70 years old compared to the 60–69 age group. Furthermore, females had an overall higher NVC compared to males (mean: 2.73 ± 1.38 vs. 2.38 ± 1.33) and demonstrated a more gradual decline across age groups, with a consistent decrease of 0.61 per cent observed between both younger than 60 and 60–69, and again between 60–69 and over 70-year-old groups. In contrast, males showed a sharper decline, with a 2.17 per cent decrease in the 60–70 age group and a further 10.2 per cent decrease in the participants over 70 years old.

### Diurnal pattern of NVC

**Figure 1:**
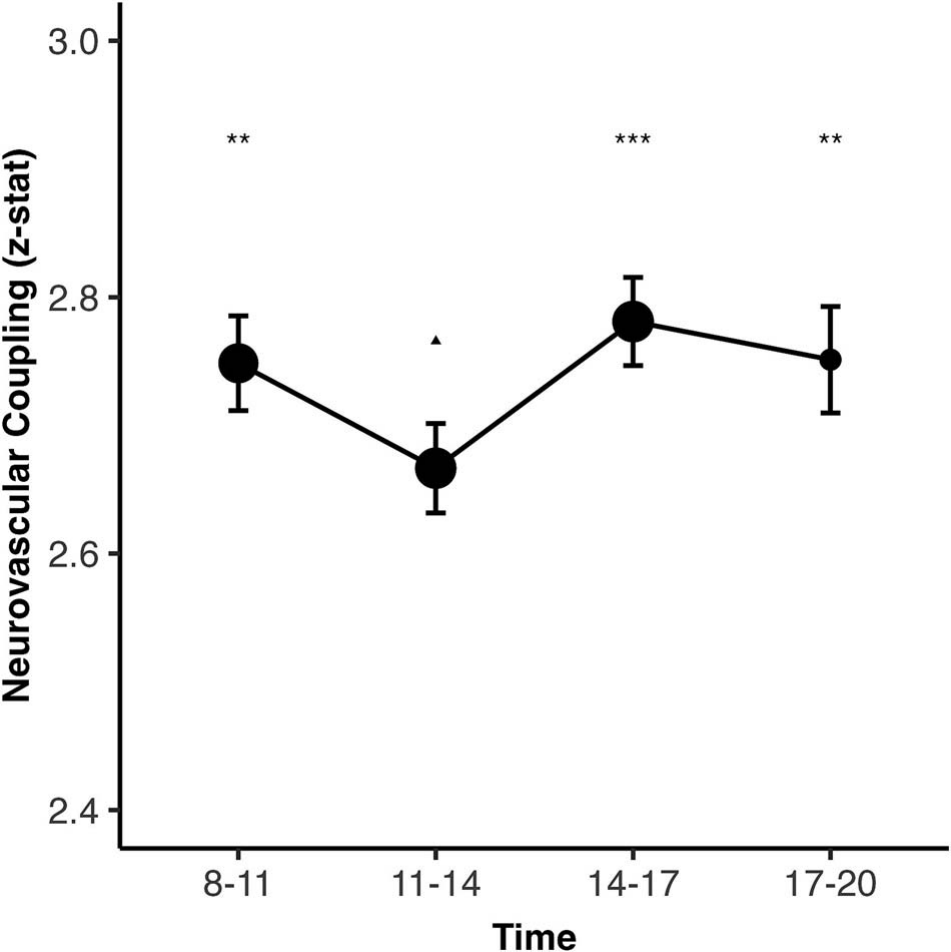
Neurovascular coupling (expressed as *z*-statistic) is presented (mean, dot size proportional to the logarithm of the subgroup size) with error bars (estimated marginal means and 95% confidence intervals, adjusted for age and sex), binned by time-of-day interval of MRI scan, with a significance difference across groups (*p* < .001, one-way ANOVA), including post hoc comparisons versus the “11–14” reference group : Tukey’s honestly significant difference (HSD) test, ^*^p < .05, ^**^p < .01, ^***^p < .001.

NVC exhibited significant diurnal variation, characterized by a dip around midday, followed by a steady rise in the afternoon (Figure 1). Age- and sex-adjusted ANOVA revealed significant differences across time (*F* = 12.12, *p* < .001). NVC was significantly lower around midday (post hoc Tukey’s HSD, 11 am to 2 pm) was significantly lower compared to all other time groups (all *p* < .01), while no statistically significant difference in NVC was present among the remaining time groups (Table S5). Similarly, when stratified into 2-h intervals, both the 10 am to 12 pm and 12 to 2 pm groups demonstrated significantly lower NVC compared to the 2 to 4 pm and 4 to 6 pm groups (Figure S3, Table S5). Notably, the observed NVC dip was not attributable to differences in age and sex between time-of-day.

Cosinor models confirmed significant NVC rhythmicity (12-h: *F*(2, 36 806) = 13.45, *p* < .001; 24-h: *F*(2, 36 806) = 4.49, *p* < .05). Cosinor modeling also revealed better fit compared to linear regression models while showing consistent significant amplitudes and acrophases in the 12-h model, underscored robust diurnal variation (Table 2, Figure S4).

**Table 2:** Time-of-day and neurovascular coupling, fitted to least squares cosine curve models assuming linear regression or period of 12 and 24 h

**Model**	**Parameters**	**Linear regression**	**Cosinor with 12-h period**	**Cosinor with 24-h period**
Unadjusted	AIC	129 331	125 848	125 866
	Amplitude	-	0.05 (0.03– 0.07)^***^	0.07 (0.01– 0.12)^*^
	Acrophase	-	2.89 (2.50– 3.29)^***^	0.07 (−0.33– 0.48)
Age and sex adjusted	AIC	128 613	125 118	125 138
	Amplitude	-	0.06 (0.04– 0.08)^***^	0.08 (0.03– 0.13)^**^
	Acrophase	-	2.95 (2.58– 3.31)^***^	0.09 (−0.24– 0.42)
Age, sex, and vascular risk factors adjusted	AIC	125 114	121 683	121 703
	Amplitude	-	0.06 (0.04– 0.08)^***^	0.08 (0.03– 0.13)^**^
	Acrophase	-	2.97 (2.60– 3.33)^***^	0.10 (−0.22– 0.42)

AIC indicates Akaike information criteria, where smaller values represent better model fit. Vascular risk factors included systolic blood pressure, diastolic blood pressure, diabetes, and waist–hip ratio. ^*^p < .05, ^**^p < .01, ^***^p < .001.

### Age, sex, and NVC

**Figure 2:**
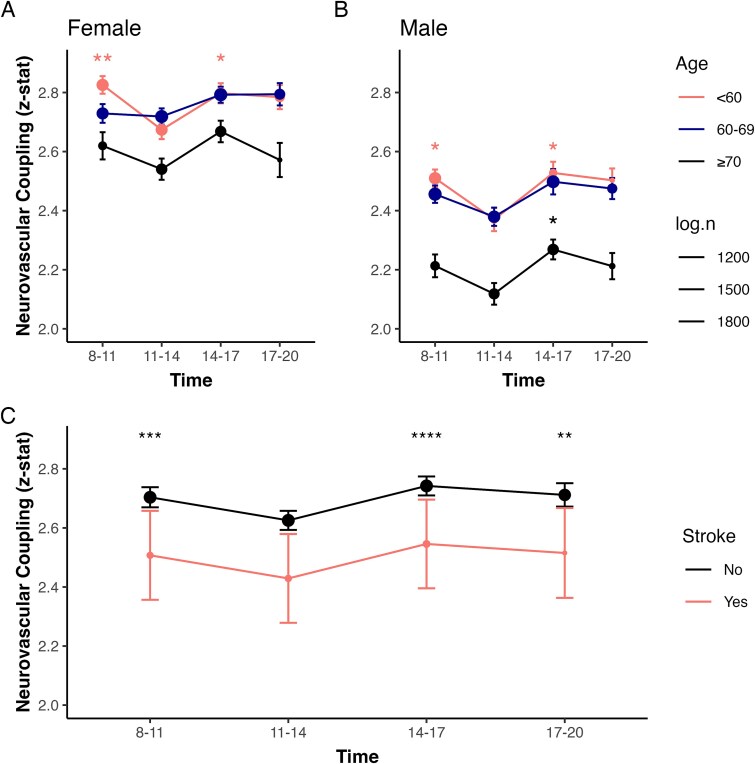
Neurovascular coupling (expressed as *z*-statistic) is presented as dots (mean, dot size proportional to the logarithm of the subgroup size) and error bars (standard error of the mean for (A and B); estimated marginal means and 95% confidence intervals, adjusted for age and sex for (C), binned by time-of-day of MRI scan, with a significance difference across groups (*p* < .001, one-way ANOVA), with post hoc comparisons versus the “11–14” reference group: Tukey’s honestly significant difference (HSD) test, ^*^p < .05, ^**^p < .01, ^***^p < .001.

When stratified by age and sex, NVC exhibited similar diurnal changes across groups (Figure 2A and B). Among females, the youngest group (<60 years) had a significant dip in NVC around midday (one-way ANOVA, *p* < .01), whereas older age groups (60–69 and ≥ 70 years) displayed relatively stable NVC throughout the day (one-way ANOVA, *p* = .15 and 0.08, respectively). In males, both younger and older subjects dipped around midday, followed by a steady rise toward the evening. These diurnal changes were consistent when stratified into 2-h time groups (Tables S6 and S7).

Cosinor analysis further confirmed significant rhythmicity in NVC for both males (12-h: *F*(2, 17 401) = 12.02; 24-h: *F*(2, 17 401) = 7.49; both *p* < .001) and females (12-h: *F*(2, 19 402) = 7.09, *p* < .001; 24-h: *F*(2, 19 402) = 3.13; both *p* < .05). When stratified by age (<60, 60–69, and ≥70 years), rhythmicity remained significant in the 12 (all *p* < .05) but not the 24-h models.

There was significant variation in amplitude and acrophase in the 12-h models across sexes and age groups, but not in the 24-h models (Figure S5, Table S8), aligned with the observed diurnal NVC patterns. However, no significant differences in amplitude or acrophase were observed between sexes (all *p* > .05, age-adjusted) or age groups (all *p* > .05, age-adjusted) in either 12-h or 24-h models.

### Cerebrovascular disease and diurnal NVC variation

Participants with a history of ischemic stroke (*N* = 363) had significantly lower overall NVC compared to those without stroke. However, no significant diurnal variation in NVC was observed within this group (one-way ANOVA, *p* = .98, fully adjusted; Figure 2C). Cosinor analysis confirmed the absence of rhythmicity (12-h: *F*(2, 360) = 0.20, *p* = .82; 24-h: *F*(2, 360) = 0.52, *p* = .60), with no significant amplitude or acrophase detected in the ischemic stroke participants (Figure S6, Table S9).

Similarly, participants with the highest SVD burden (quartile Q4 of nWMH volume) exhibited significantly lower overall NVC (Figure S7). Despite this reduction, diurnal rhythmicity in NVC remained intact across all SVD burden subgroups. Significant rhythmicity, amplitude, and acrophase were observed in the 12-h cosinor models (Table S10), further supporting the persistence of diurnal NVC variation even in the presence of macrostructural cerebrovascular pathology.

### Sleep quality, night shift work, and NVC

**Figure 3:**
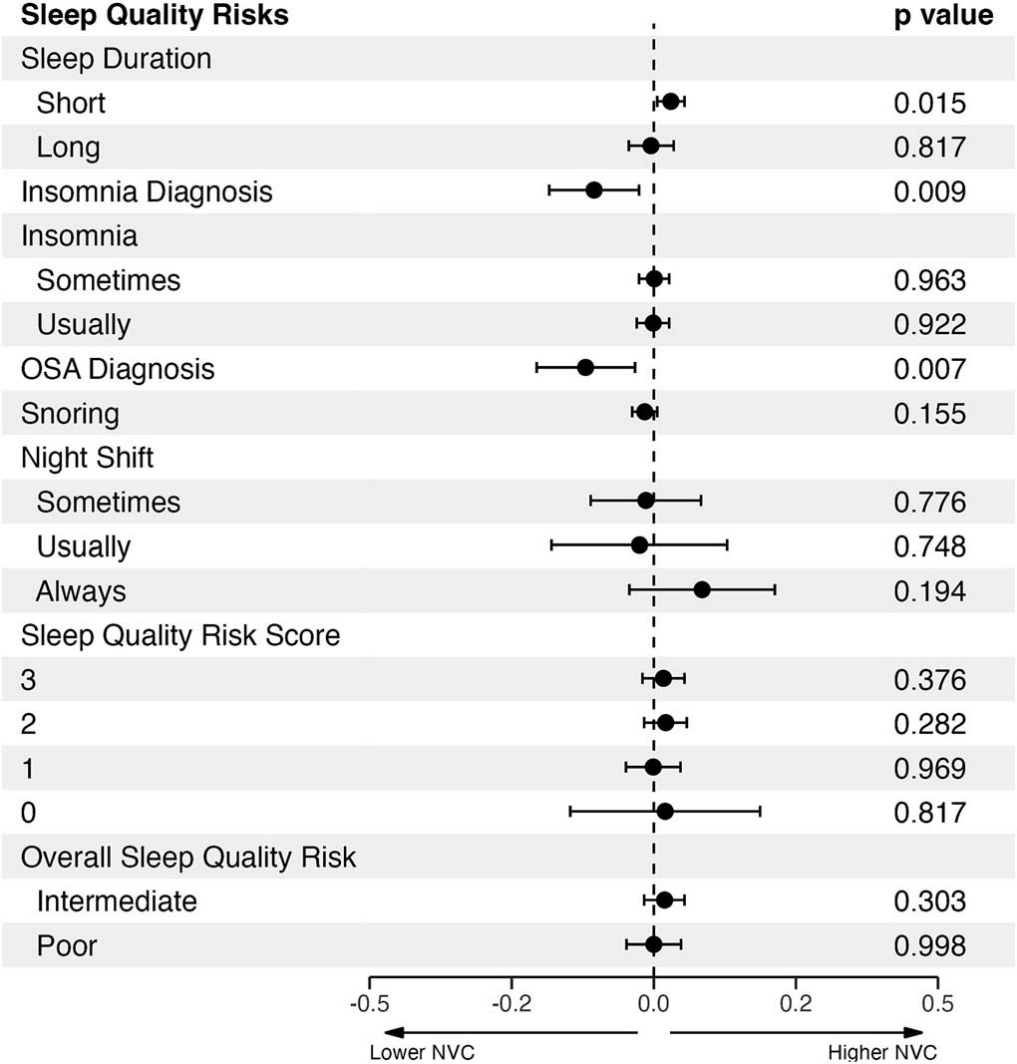
Associations between neurovascular coupling (NVC) and individual sleep quality risk factors is presented as standardized coefficients (*β*) with 95% confidence intervals, derived from general linear models adjusted for age, sex, and vascular risk factors, comparing sleep duration inshort (<7 h), optimal (7–8 h), and long (≥9 h) groups compared to the reference groups: “Normal” sleep duration, reporting “No/Never” to obstructive sleep apnea (OSA), snoring, insomnia, night shifts, highest sleep quality risk score, and “Healthy” overall sleep quality risk.

Compared to individuals with recommended sleep length (7–8 h), those with shorter sleep (<7 h) had significantly higher overall NVC, while there was no significant difference in NVC between recommended sleep length and those who slept longer than 8 h. There was no significant difference between subjects who did or did not work night shifts (Figure 3).

Diagnosed sleep apnea and insomnia were significantly associated with lower NVC in fully adjusted models. Similarly, snorers had significantly lower NVC (*β* = −0.05 [95% CI = −0.07 to −0.03], *p* < .001), though this effect was diminished after controlling for age, blood pressure, and vascular risks. However, individuals self-reporting insomnia “sometime” or “usually” had higher NVC than those reporting less-frequent insomniac episodes, though this relationship did not persist after adjustments. Poorer overall sleep pattern (lower Sleep Quality Risk Score) tended to be associated with lower NVC compared to healthy sleep pattern, though this was not statistically significant (Figure 3).

**Figure 4:**
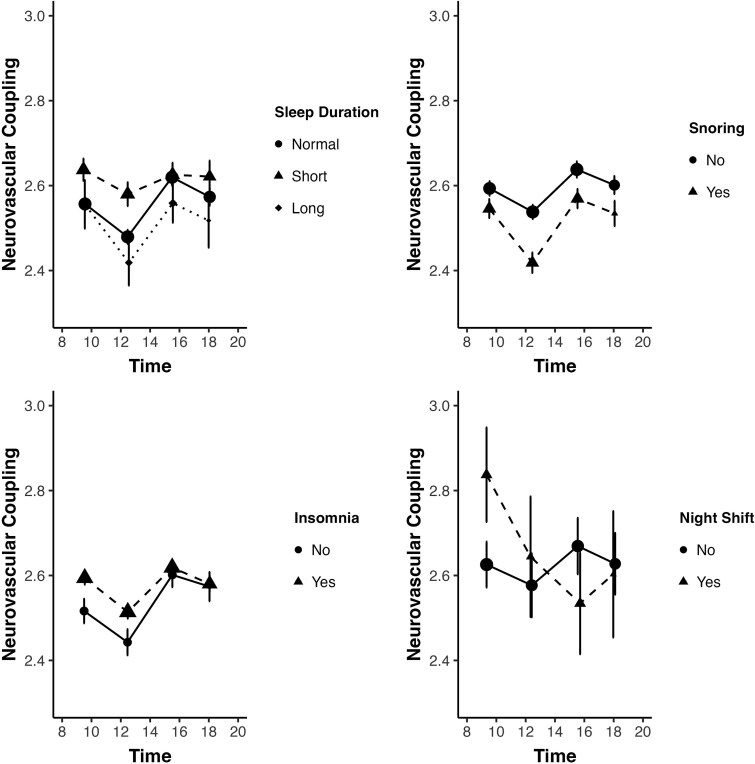
Neurovascular coupling (expressed as *z*-statistic) is presented as dots and error bars (mean ± standard error of the mean), comparing groups by time-of-day of MRI scan, with a significance difference across groups (*p* < .001, one-way ANOVA), with post hoc comparisons versus the “11–14” reference group by Tukey’s honestly significant difference (HSD) test, ^*^p < .05, ^**^p < .01, ^***^p < .001.

In examining the effects of sleep quality risk factors on the rhythmicity of NVC, subjects with healthy sleep patterns (normal sleep duration, no history of night shift work or insomnia, no sleep apnea, or snoring) showed a consistent midday dip in NVC. Poorer sleep patterns showed no significant effects on the fluctuations of NVC. However, night shift workers had a delayed dip in NVC, occurring at around 4 pm (Figure 4, Table S11).

## Discussion

In the UKB study, cerebrovascular function exhibited a diurnal pattern, with a history of stroke and individual sleep quality risk factors affecting the absolute mean value of BOLD signal but having less effect on its rhythmicity. NVC, indicated by a standardized change in BOLD signal in response to a simple visual stimulus and reflected cerebral endothelial function, fluctuated throughout the day with a notable midday dip. These findings suggest that endothelial vasodilatory capacity may affect resilience to acute ischemia, and its disturbance due to impaired sleep could impact upon the timing of and probability of strokes [2, 23, 49].

### Diurnal variation in NVC

NVC mediates regional blood flow to neuronal demand, and cellular components of the neurovascular unit display endogenous circadian variations [1, 49]. This study showed that the magnitude of NVC has a predominant diurnal fluctuation with a consistent midday NVC dip observed on a 12-h cycle rather than a 24-h cycle, but these rhythms do not differ significantly by age or sex. A recent review [12] by Webb et al. on the diurnal variations in cerebrovascular function in human summarized four indexes of cerebral vascular function: CBF, CVR, cerebral autoregulation (CA), and NVC. Transcranial Doppler was most commonly used to measure CBF, CVR, and CA, while BOLD-fMRI was primarily for NVC. Reduced CBF and CBF velocity were observed at night in some studies, while others showed stable CBF throughout the day. In comparison, there is a lack of evidence on CA and NVC, with sporadic studies with small sample sizes showing inconsistent results.

Diurnal variations in vascular tone and reactivity reflect the integration between ubiquitous diurnal variations in brain function under the primary influence of the master clock in the suprachiasmatic nucleus with external bright and dark conditions and endogenous cellular rhythms [50]. Measured diurnal variations in neural activation may therefore reflect not only variations in the neural component of responses, but also in vascular functional effects as well, for example with reduced responses to food stimuli during the evening in the left putamen, a region involved in reward processing [51], brain volume [52], and resting-state fMRI [53].

### Cerebrovascular disease impacted NVC’s diurnal change

Participants with a history of ischemic stroke exhibited both lower overall NVC and a loss of diurnal rhythmicity, whereas those with a high SVD burden demonstrated reduced NVC but retained significant diurnal variation. While both conditions share common risk factors and contribute to cerebrovascular dysfunction, their effects on NVC rhythmicity suggest distinct underlying mechanisms.

A history of ischemic stroke reduced overall NVC and rhythmicity in this study, with potential loss of the midday dip, although this could have been an artifact of the size of the stroke group. This may reflect widespread, long-term vascular dysfunction, and impaired cerebrovascular adaptability following an acute vascular event. This is supported by epidemiological findings that showed a morning surge in the incidence of ischemic strokes, transient ischemic attacks, and hemorrhagic strokes, with peak occurrence generally observed between 6 am and 12 noon across stroke subtypes [2, 12, 54], including large artery atherosclerotic, cardioembolic, and SVD-related strokes.

In contrast, SVD is characterized by a chronic progressive change in the microvasculature, including endothelial dysfunction, blood–brain barrier disruptions, and impaired vasoreactivity. Despite its impact on overall vascular function, the endothelium may retain some capacity to adapt to circadian rhythms.

### Impact of sleep quality on NVC

Of the sleep quality risk factors examined, OSA stood out as an independent negative factor associated with reduced NVC. This is particularly noteworthy given the well-established relationship between OSA and increased stroke risk [55, 56], as well as the beneficial effects of airway management therapy, such as particular continuous positive airway pressure (CPAP), in improving OSA symptoms and reducing stroke risk [57]. OSA has also been linked to decreased CVR, with CPAP therapy shown to improve CVR in persons with OSA [58, 59], supporting the connection between endothelial dysfunction and pathogenesis of OSA [60]. This could therefore reflect either disturbance of synchronization of the circadian clocks due to disturbed nocturnal sleep, or a direct vascular effect, for example due to desensitization of vasodilatory pathways to stimulation due to intermittent nocturnal hypercapnia. Future studies or clinical trials could explore in greater depth the effects of OSA on NVC and investigate whether airway management can mitigate some of the adverse impacts on NVC.

The positive correlation of short sleep period with NVC is in contrast to shorter sleep being associated with increased blood pressure [61], cardiovascular events [62, 63], and endothelial dysfunction [64]. This result may reflect selection bias in healthier short-sleeping individuals, limitations of self-reported sleep, or compensatory vascular mechanisms. Further studies using objective sleep tracking are needed to clarify the specific sleep duration thresholds that promote optimal NVC. The relationship between insomnia and NVC was more intricate. While a history of insomnia was significantly associated with lower NVC, individuals reporting experiencing insomnia had similar NVC compared to those without, though this may reflect either missing data effects (only 5.8 per cent of participants in this study had self-reported insomnia data in UKB) or confounders in reporting of experienced sleep disturbance that was discordant with actual sleep duration.

Interestingly, the study did not find a significant association between night shift work and overall NVC [65]. This contrasts with some previous literature suggesting that shift work disrupts circadian rhythms and negatively affects cardiovascular health [3, 61]. However, the limited number of participants reporting frequent night shift work (5.8 per cent) may have reduced the power to detect such associations. Notably, among those who did report night shift work, the midday dip in NVC appeared delayed, occurring closer to the late afternoon. This shift in diurnal pattern may reflect alterations in the circadian system or other physiological adaptations related to disrupted sleep–wake schedules, warranting further investigation in cohorts with more detailed chronotype and shift work data.

### Implications for future work

The diurnal pattern of NVC underscores the need for further research into the mechanisms underlying diurnal variations in cerebrovascular function, with one possible aim of developing targeted interventions that enhance cerebrovascular resilience at vulnerable times of the day. For instance, the timing of stroke prevention strategies, such as antihypertensive medication administration, might be optimized by aligning with periods of peak cerebrovascular vulnerability. Additionally, more studies are needed on sleep quality and NVC, especially for those at risk for cerebrovascular diseases.

### Limitations

There are several limitations to this study. Firstly, the timing of the brain scans, restricted to the period between 8 am and 8 pm, does not capture potential variations in NVC across the full 24-h circadian cycle. While cosinor modeling enabled the identification of rhythmic variation during waking hours, patterns beyond this observed window cannot be inferred. Future studies should aim to include a more comprehensive 24-h analysis. Secondly, the cross-sectional design limits causal inference between sleep and NVC. Thirdly, the study relied on self-reported measures of sleep quality, which may be subject to recall bias, and the sleep risk score's validity outside the UKB needed further investigation. Fourthly, information on wake time, daytime napping, sleep patterns, or night shift work immediately prior to scanning was unavailable. Participants were not instructed to modify their sleep or work schedules, so acute circadian misalignment may have influenced NVC in some cases. Fifthly, information on participants’ medication use, in particular antihypertensives, antidepressants, or other agents that may influence cerebrovascular function and sleep quality, was not available in this analysis, limiting the ability to account for pharmacological effects on NVC. Sixthly, UKB protocol did not include collection of classic circadian research markers, such as melatonin or cortisol. Finally, missing data, particularly on night shifts, weakened this analysis. Future studies with longitudinal designs, using more objective and validated sleep monitoring measures, blood markers, and could address these limitations and strengthen the evidence for causal relationships between sleep and NVC.

## Conclusion

This study demonstrated diurnal variation in NVC, that this temporal pattern could be disrupted in cerebrovascular disease, and poorer sleep quality’s impact on cerebrovascular function. These findings strengthened the implications for the timing of clinical interventions and underscored the importance of addressing sleep disorders in the prevention of cerebrovascular diseases. Investigating the long-term effects of sleep habits, particularly insufficient sleep and shift work, on cerebrovascular functions is also crucial. Further research is needed to elucidate the mechanisms driving these diurnal patterns and to explore potential therapeutic strategies to enhance overall brain health.

## Supplementary Material

Supplementary_materials_zsaf256

STROBE-checklist_zsaf256

## Data Availability

This research utilized data from the UK Biobank (application #41213, https://www.ukbiobank.ac.uk/). Ethical approval for UK Biobank data collection was granted by the North West Multi-Centre Research Ethical Committee, adhering to the principles of the 1975 Declaration of Helsinki. All participants provided written informed consent. Access to UK Biobank data is available to approved researchers via application.
