# Supplementary material for: Diurnal variation in neurovascular coupling and the impact of sleep quality: a UK Biobank study
Source: Sleep. 2025 Aug 29;49(3):zsaf256. doi: 10.1093/sleep/zsaf256 (PMC13017620; doi:10.1093/sleep/zsaf256)
Supplement: Supplementary_materials_zsaf256 [file supplementary_materials_zsaf256.docx]

**Supplementary Materials**

Title: Diurnal variation in neurovascular coupling and the impact of sleep quality: a UK Biobank study

Authors: Sheng Yang^1^ and Alastair John Stewart Webb^1,2^

Author affiliations:

1. Wolfson Centre for Prevention of Stroke and Dementia, Nuffield Department of Clinical Neurosciences, University of Oxford, Oxford, OX3 9DU, United Kingdom

2. Imperial College London, Department of Brain Sciences, Burlington Danes Building, London, London, W12 0NN, United Kingdom

Correspondence to: Alastair Webb

Full address: Imperial College London, Department of Brain Sciences, Burlington Danes Building, London, London, W12 0NN, United Kingdom

E-mail: [alastair.webb@imperial.ac.uk](mailto:alastair.webb@imperial.ac.uk)

Table of Contents

[Supplementary Figure 1. Flowchart for selection of subjects. 3](#_Toc206853475)

[Supplementary Figure 2. Normality test for continuous variables. 4](#_Toc206853476)

[Supplementary Figure 3. NVC changes with time-of-day. 5](#_Toc206853477)

[Supplementary Figure 4. Time-of-day and NVC, fitted to least squares cosine curve models assuming period of 12 and 24-hour period, adjusted for age, sex and vascular risk factors. 6](#_Toc206853478)

[Supplementary Figure 5. Time-of-day and NVC, fitted to least squares cosine curve models assuming period of 12 and 24-hour period, stratified by age and sex. 7](#_Toc206853479)

[Supplementary Figure 6. Time-of-day and NVC, fitted to least squares cosine curve models assuming period of 12 and 24-hour period, adjusted for age, sex and vascular risk factors, stratified by ischaemic stroke history. 8](#_Toc206853480)

[Supplementary Figure 7. Time-of-day and NVC, fitted to least squares cosine curve models assuming period of 12 and 24-hour period, adjusted for age, sex and vascular risk factors, stratified by cerebral small vessel disease burden (logged white matter hyperintensities volume). 9](#_Toc206853481)

[Supplementary Table 1. UK Biobank MRI image acquisition protocols and pipeline of selected image-derived phenotypes. 10](#_Toc206853482)

[Supplementary Table 2. UK Biobank data included in this study. 11](#_Toc206853483)

[Supplementary Table 3a. Demographics of female and male subjects. 13](#_Toc206853484)

[Supplementary Table 3b. Demographics of subjects with a history of ischaemic stroke. 14](#_Toc206853485)

[Supplementary Table 4. Demographics of SVD burden groups (normalised WMH). 15](#_Toc206853486)

[Supplementary Table 5. Tukey post-hoc analysis of NVC difference between time-of-day groups, adjusted for age and sex. 16](#_Toc206853487)

[Supplementary Table 6. Tukey post-hoc analysis of NVC difference between time-of-day groups (3-hour bins), stratified by age and sex. 17](#_Toc206853488)

[Supplementary Table 7. Tukey post-hoc analysis of NVC difference between time-of-day groups (2-hour bins), stratified by age and sex. 18](#_Toc206853489)

[Supplementary Table 8. Cosinor analysis. 20](#_Toc206853490)

[Supplementary Table 9. Cosinor analysis of stroke. 20](#_Toc206853491)

[Supplementary Table 10. Cosinor analysis of cerebral small vessel disease burden. 20](#_Toc206853492)

[Supplementary Table 11. Tukey post-hoc analysis of NVC difference between time-of-day groups, stratified by sleep duration, adjusted for age, sex and vascular risk factors. 21](#_Toc206853493)

[Reference 22](#_Toc206853494)

## Supplementary Figure 1. Flowchart for selection of subjects.


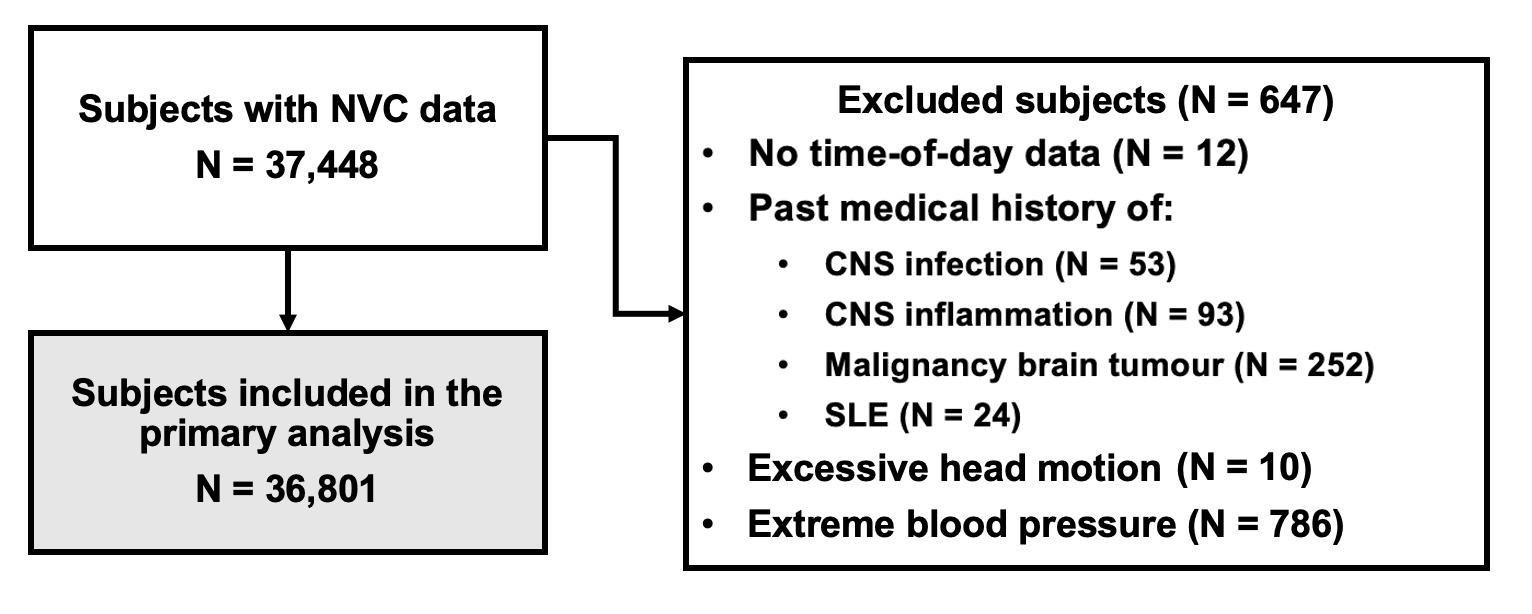


## Supplementary Figure 2. Normality test for continuous variables.

NVC: neurovascular coupling, SBP: systolic blood pressure, DBP: diastolic blood pressure, WHR: waist-hip ratio, nWMH: normalised white matter hyperintensity


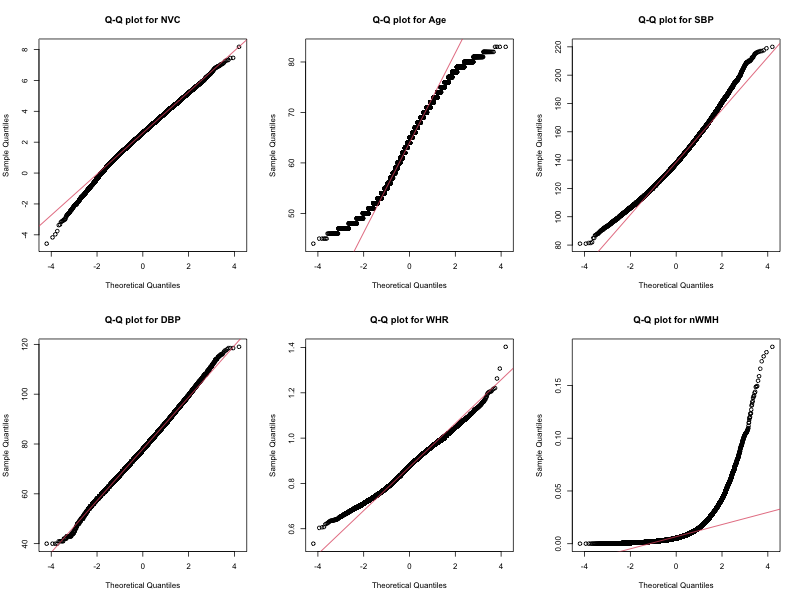


## Supplementary Figure 3. NVC changes with time-of-day.

Neurovascular coupling (expressed as z-statistic) is presented as dots (mean, dot size proportional to the logarithm of the subgroup size) and error bars (estimated marginal means and 95% confidence intervals, adjusted for age and sex), binned by time-of-day of MRI scan, with a significant difference across groups (p < 0.001, one-way ANOVA). Post-hoc comparisons versus the “10-12” (denoted by *) and “12-14” (denoted by +) groups are shown: Tukey’s honestly significant difference (HSD) test, */+ p < 0.05, **/++ p < 0.01, ***/+++ p < 0.001.


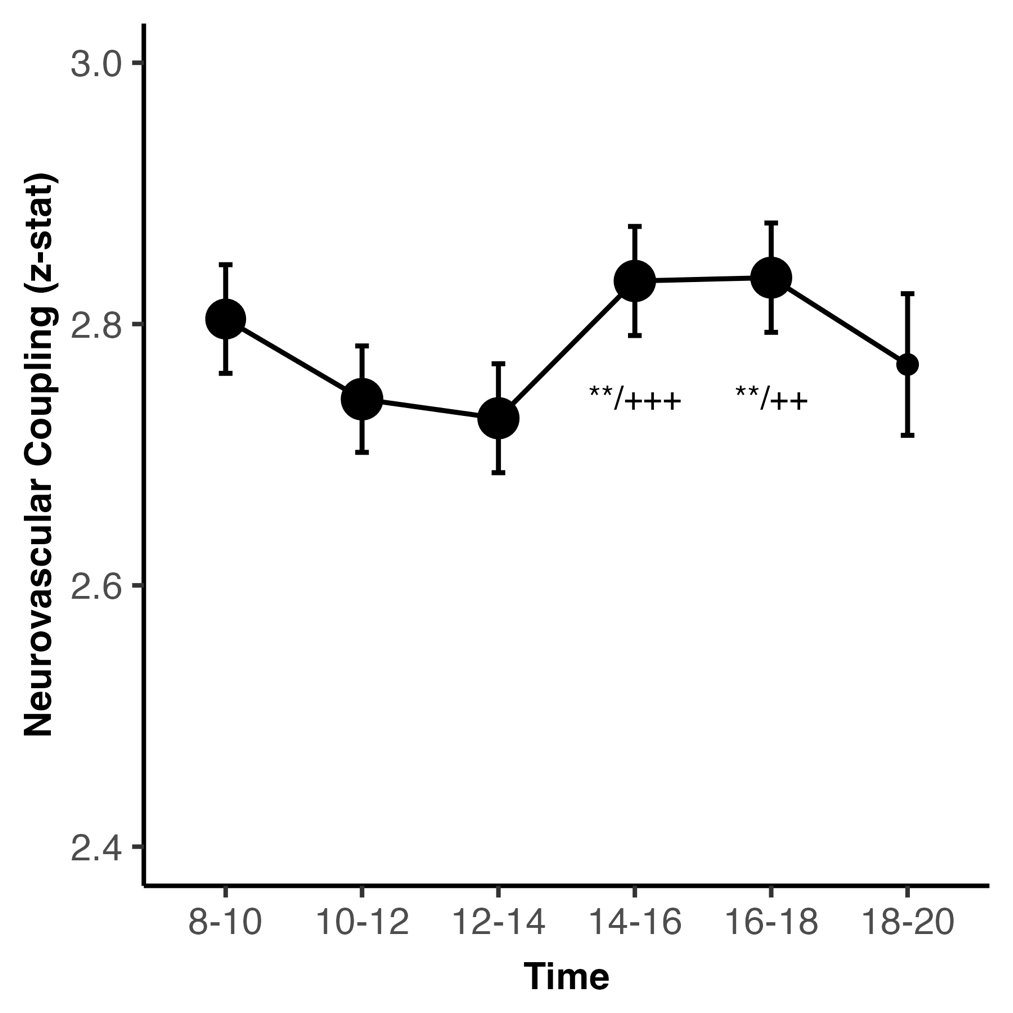


## Supplementary Figure 4. Time-of-day and NVC, fitted to least squares cosine curve models assuming period of 12 and 24-hour period, adjusted for age, sex and vascular risk factors.


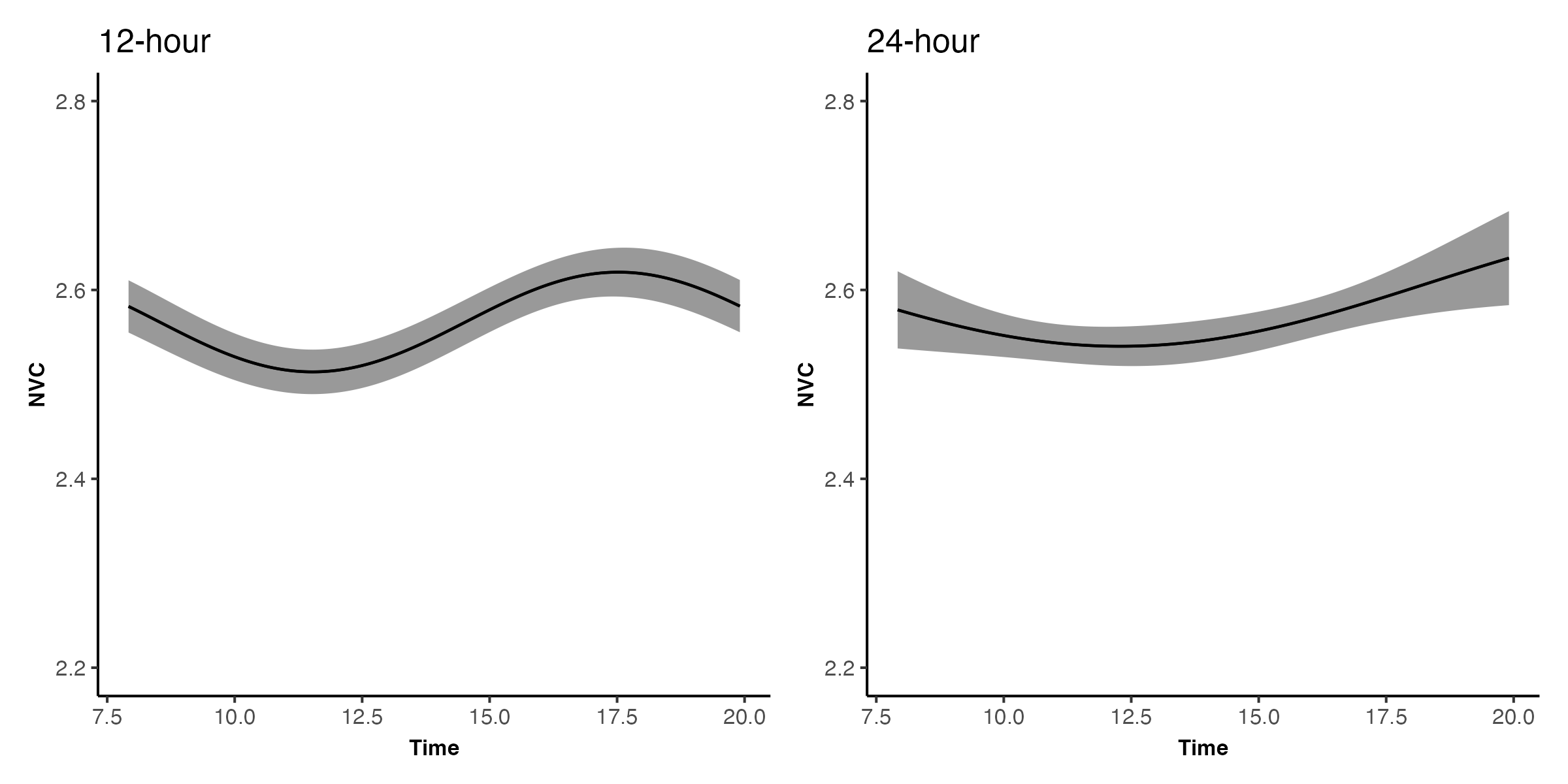


## Supplementary Figure 5. Time-of-day and NVC, fitted to least squares cosine curve models assuming period of 12 and 24-hour period, stratified by age and sex.

Numbers of each subgroup (N): Female <60 (6329), 60-69 (8465), ≥70 (4611); Male <60 (4816), 60-69 (7221), ≥70 (5359).


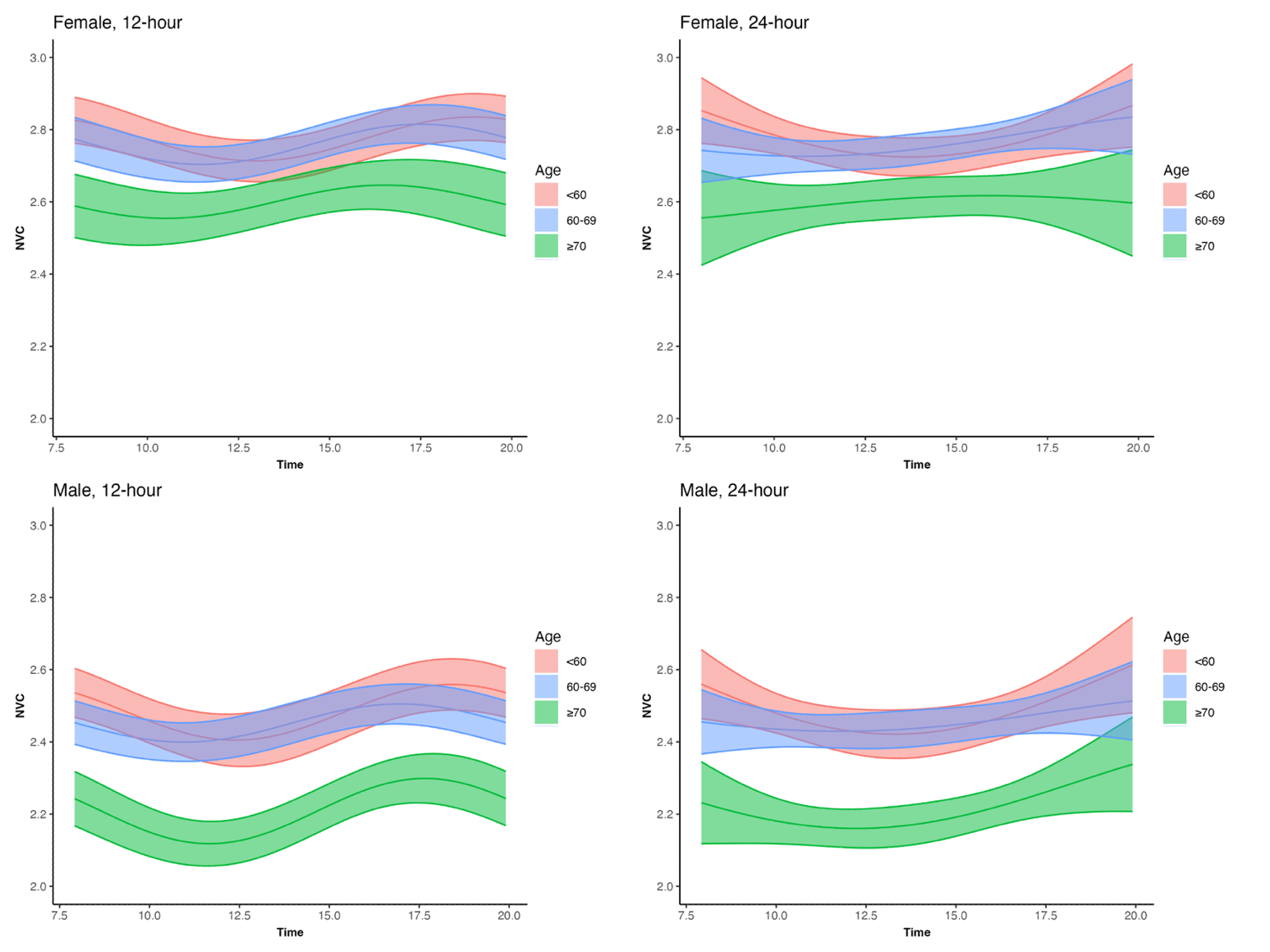


## Supplementary Figure 6. Time-of-day and NVC, fitted to least squares cosine curve models assuming period of 12 and 24-hour period, adjusted for age, sex and vascular risk factors, stratified by ischaemic stroke history.

Numbers of each subgroup (N): participants with ischaemic stroke history (363), without ischaemic stroke (36438).


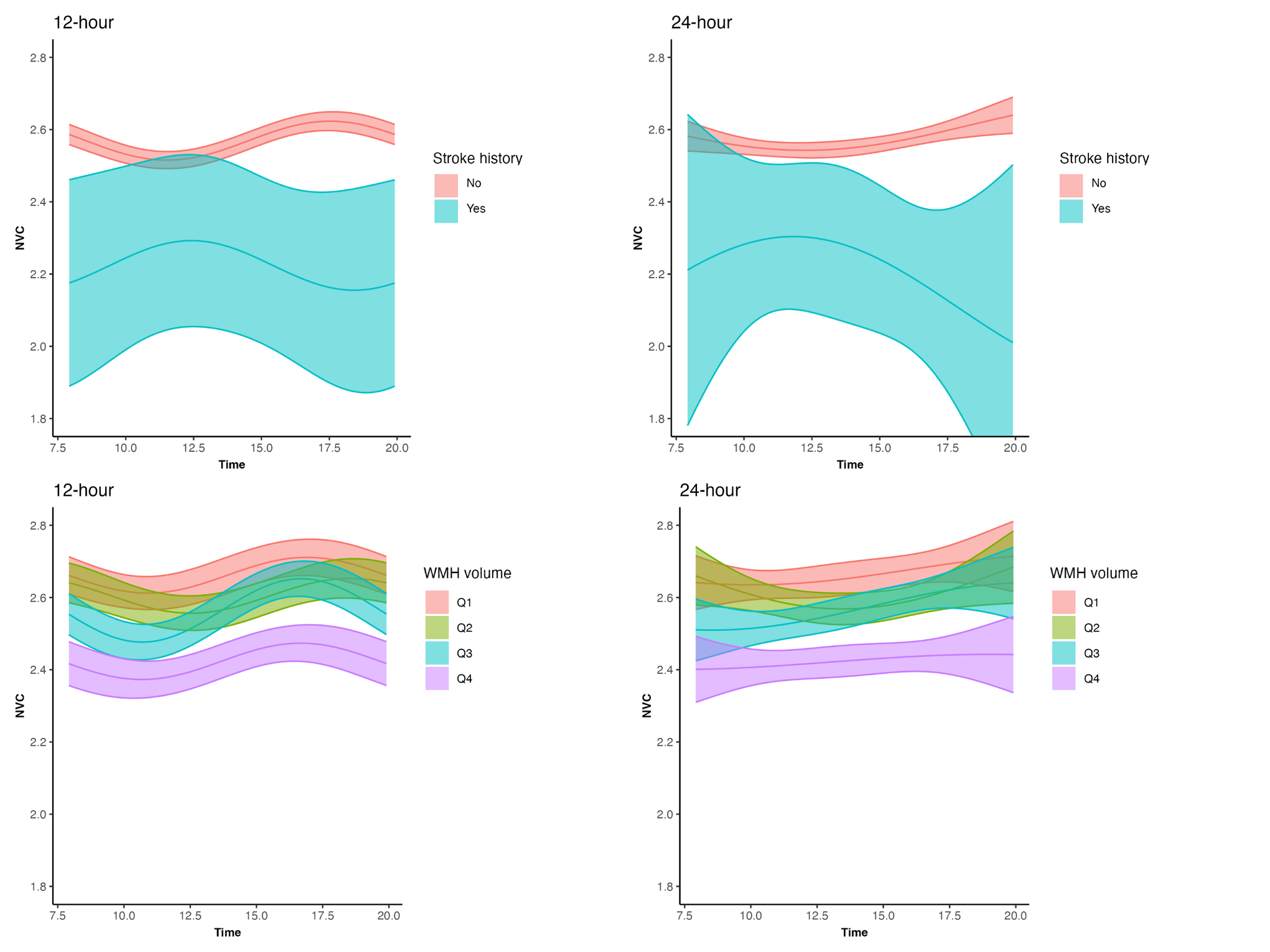


## Supplementary Figure 7. Time-of-day and NVC, fitted to least squares cosine curve models assuming period of 12 and 24-hour period, adjusted for age, sex and vascular risk factors, stratified by cerebral small vessel disease burden (logged white matter hyperintensities volume).

Numbers of each subgroup (N): Q1 (9061), Q2 (9061), Q3 (9063), Q4 (9061).


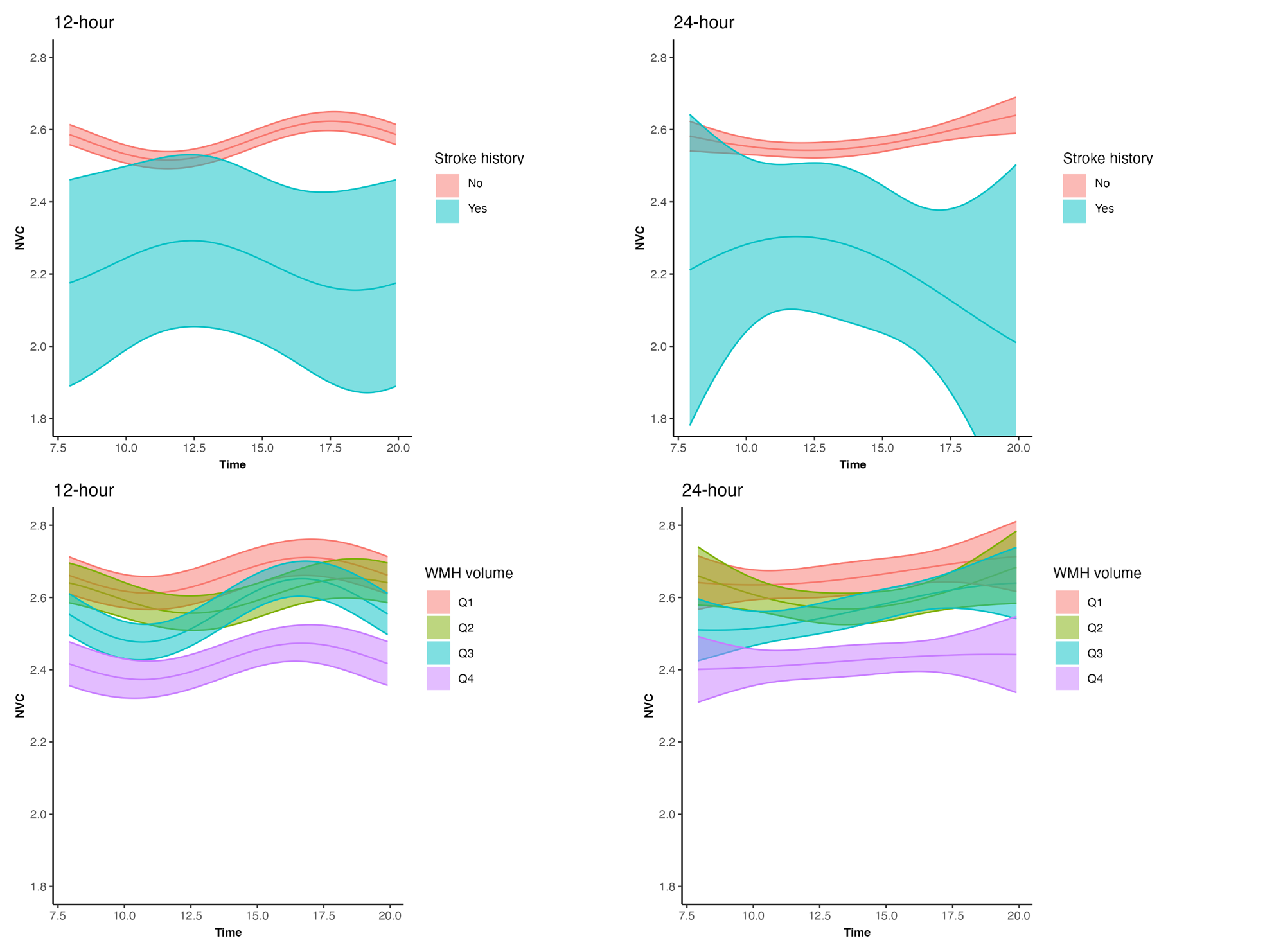


## Supplementary Table 1. UK Biobank MRI image acquisition protocols and pipeline of selected image-derived phenotypes.

The full details of UKB brain MRI acquisition methods can be found in the primary brain imaging documentation: <http://biobank.ctsu.ox.ac.uk/crystal/crystal/docs/brain_mri.pdf>. The UKB obtained brain MRI data using a Siemens Skyra 3 Tesla scanner, and the parameters for the sequence have been previously published.^1^ The UKB brain imaging team utilized FMRIB Software Library (FSL) tools (http://www.fmrib.ox.ac.uk/fsl) to pre-process and analyse T1-weighted 3D magnetization-prepared 180 degrees radiofrequency pulses, rapid gradient-echo, and fMRI data. The analysis pipeline and quality assessment have been previously outlined,^2^ wherein the T1-weighted images underwent skull-stripping and bias field correction with the Brain Extraction Tool,^3^ followed by segmentation into white and grey matter and cerebrospinal fluid using FMRIB’s Automated Segmentation Tool (FAST).^4^ Image-derived phenotypes (IDPs) representing objective quantifications of different aspects of brain structure and function were generated, and the pipeline was previously published.^2^

| **Modality** | **Description** | |
| --- | --- | --- |
| T1-weighted structural imaging | Acquisition  Protocols | - Resolution: 1x1x1 mm - Field-of-view: 208x256x256 matrix - Duration: 5 minutes - 3D MPRAGE, sagittal, in-plane acceleration iPAT=2, prescan-normalise |
|  | IDPs | - Volume of brain, grey + white matter (normalized for head size) (**Data-field #25009**) |
| T2-weighted FLAIR structural imaging | Acquisition  Protocols | - Resolution: 1.05x1x1 mm - Field-of-view: 192x256x256 matrix - Duration: 6 minutes - 3D SPACE, sagittal, in-plane acceleration iPAT=2, partial Fourier=7/8, fat saturation, elliptical k-space scanning, prescan-normalise |
|  | IDPs | - Total volume of white matter hyperintensities^5^ (from T1 and T2_FLAIR images) (**Data-field #25781**) calculated with BIANCA (Brain Intensity AbNormality Classification Algorithm).^6^ The total volume of white matter hyperintensities was divided by brain volume to get normalized white matter hyperintensities (nWMH). |

## Supplementary Table 2. UK Biobank data included in this study.

|  | **Data-fields** | **Description** |
| --- | --- | --- |
| **Image-derived phenotypes** | | |
| Structural MRI | Acquisition protocols | Summarized in **Table S1** |
|  | 25009 | Volume of brain, grey + white matter (normalized for head size) |
|  | 25781 | Total volume of white matter hyperintensities (from T1 and T2_FLAIR images) |
| Functional MRI | Acquisition protocols | Summarized in **Table S1** |
|  | 20249 | Task-related functional brain images (NIFTI) |
|  | 25042 | Median z-statistic (in group-defined mask) for shapes activation |
|  | 25742 | Mean tfMRI head motion, averaged across space and time points |
| **Vascular risk factors** | | |
| Unmodifiable factors | 21003 | Age |
|  | 31 | Sex |
|  | 21000 | Ethnic background |
| Body size measures | Procedure for body composition measurement | https://biobank.ctsu.ox.ac.uk/crystal/ukb/docs/body_composition.pdf |
|  | 48 | Waist circumference |
|  | 49 | Hip circumference |
| Blood pressure measures | Procedure for blood pressure measurement | https://biobank.ctsu.ox.ac.uk/crystal/ukb/docs/Bloodpressure.pdf |
|  | 4080 | Systolic blood pressure, automated reading |
|  | 93 | Systolic blood pressure, manual reading |
|  | 12674 | Systolic brachial blood pressure during pulse wave analysis |
|  | 4081 | Diastolic blood pressure, automated reading |
|  | 94 | Diastolic blood pressure, manual reading |
|  | 12675 | Diastolic brachial blood pressure during pulse wave analysis |
| Disease measures | 41270 | Diagnoses – ICD10 |
| **Sleep quality risk factors (self-reported)** | | |
|  |  | Participants reported snoring as "yes" or "no"; the frequency of daytime sleepiness, insomnia, and night shift were reported as "never/rarely," "sometimes," or "usually," with night shift including an additional option of "always"; sleep duration was reported as positive integer; sleep chronotype was reported as “morning person,” “leaning towards morning,” “leaning towards evening,” or “evening person.” |
|  | 1210 | Snoring |
|  | 1200 | Insomnia |
|  | 1160 | Sleep duration |
|  | 1180 | Sleep chronotype |
|  | 3246 | Night shift |
| **Health-related outcomes** | | |
| Stroke outcomes | 42006 | Date of stroke |
|  | 42008 | Date of ischaemic stroke |
|  | 42010 | Date of intracerebral haemorrhage |
|  | 42012 | Date of subarachnoid haemorrhage |
| **ICD-10 codes** | | |
| Conditions for exclustion | C71 | Malignant neoplasm of brain |
|  | G00-G09 | Inflammatory diseases of the central nervous system |
|  | I60 | Subarachnoid haemorrhage |
|  | I61, I62 | Intracerebral haemorrhage, other nontraumatic intracranial haemorrhage |
|  | I63 | Cerebral infarction |
|  | M32 | Systemic lupus erythematosus |
|  | E10-E14 | Diabetes mellitus |
| Sleep quality risk factors | G47.33 | Obstructive sleep apnoea |
|  | G47.0 | Insomnia |

## Supplementary Table 3a. Demographics of female and male subjects.

Continuous variables are shown as mean (SD) and categorical variables as count (%).

| **Variable** | **Total** | **Female** | **Male** | **P value** |
| --- | --- | --- | --- | --- |
| **N** | 36801 | 19405 | 17396 | <0.001 |
| **Age (years)** | 63.99 (7.74) | 63.38 (7.59) | 64.67 (7.85) | <0.001 |
| **White (N)** | 33617 (91.3%) | 17751 (91.5%) | 15866 (91.2%) | <0.001 |
| **SBP (mmHg)** | 139.16 (18.57) | 136.91 (19.45) | 141.67 (17.19) | <0.001 |
| **DBP (mmHg)** | 78.00 (10.45) | 76.28 (10.46) | 79.92 (10.09) | <0.001 |
| **Waist-hip ratio** | 0.88 (0.09) | 0.82 (0.07) | 0.94 (0.06) | <0.001 |
| **Normalized WMH volume^a^** | 0.01 (0.01) | 0.01 (0.01) | 0.01 (0.01) | <0.001 |
| **Has ischaemic stroke history (N)** | 363 (1.0%) | 126 (0.6%) | 237 (1.4%) | <0.001 |
| **Sleep quality risk score^b^** | 2.47 (0.82) | 2.45 (0.78) | 2.48 (0.89) | <0.05 |
| **Sleep duration (N)^c^** |  | | | |
| Normal | 24992 (67.9%) | 12978 (67.5%) | 12014 (69.6%) | <0.001 |
| Short | 8850 (24.0%) | 4995 (26.0%) | 3855 (22.3%) |  |
| Long | 2643 (7.2%) | 1242 (6.5%) | 1401 (8.1%) |  |
| **Insomnia (N)^d^** |  | | | |
| Never/rarely | 7963 (21.6%) | 3028 (15.7%) | 4935 (28.6%) | <0.001 |
| Sometimes | 16831 (45.7%) | 9288 (48.2%) | 7543 (43.7%) |  |
| Usually | 11756 (31.9%) | 6958 (36.1%) | 4798 (27.8%) |  |
| **Insomnia diagnosis (N)** | 618 (1.7%) | 179 (9.2%) | 439 (25.2%) | <0.001 |
| **OSA diagnosis (N)** | 518 (1.4%) | 128 (0.7%) | 390 (2.2%) | <0.001 |
| **Snorers (N)** | 12278 (33.4%) | 5052 (26.0%) | 7226 (41.5%) | <0.001 |
| **Night shift work (N) ^e^** |  | | | |
| Never/rarely | 1117 (52.5%) | 606 (59.4%) | 511 (46.1%) | <0.001 |
| Sometimes | 574 (26.9%) | 246 (24.1%) | 328 (29.6%) |  |
| Usually | 169 (7.9%) | 69 (6.8%) | 100 (9.0%) |  |
| Always | 269 (12.6%) | 100 (9.8%) | 169 (15.3%) |  |

SBP: systolic blood pressure; DBP: diastolic blood pressure; WMH: white matter hyperintensity; OSA: obstructive sleep apnoea.

Data was available in a 98.5%, b 90.2%, c 99.1%, d 99.3%, e 5.8%, of data, respectively.

## Supplementary Table 3b. Demographics of subjects with a history of ischaemic stroke.

Continuous variables are shown as mean (SD) and categorical variables as count (%).

|  |  | **History of Ischaemic Stroke** | |  |
| --- | --- | --- | --- | --- |
| **Variable** | **Total** | **Yes** | **No** | **P value** |
| **N** | 36801 | 363 | 36438 | <0.001 |
| **Age (years)** | 63.99 (7.74) | 68.25 (7.13) | 63.94 (7.73) | <0.001 |
| **Male (N)** | 17396 (47.3%) | 237 (65.3%) | 17159 (47.1%) | <0.001 |
| **White (N)** | 33617 (91.3%) | 332 (91.5%) | 33285 (91.3%) | <0.001 |
| **SBP (mmHg)** | 139.16 (18.57) | 144.83 (21.02) | 139.10 (18.53) | <0.001 |
| **DBP (mmHg)** | 78.00 (10.45) | 79.34 (11.97) | 77.99 (10.43)) | <0.001 |
| **Waist-hip ratio** | 0.88 (0.09) | 0.91 (0.08) | 0.87 (0.09) | <0.001 |
| **Normalized WMH volume^a^** | 0.01 (0.01) | 0.01 (0.02) | 0.01 (0.01) | <0.001 |
| **Sleep quality risk score^b^** | 2.47 (0.82) | 2.51 (0.82) | 2.46 (0.82) | 0.35 |
| **Sleep duration (N)^c^** |  | | | |
| Normal | 24992 (67.9%) | 229 (64.5%) | 24763 (68.5%) | <0.001 |
| Short | 8850 (24.0%) | 74 (20.8%) | 8776 (24.3%) |  |
| Long | 2643 (7.2%) | 52 (14.6%) | 2591 (7.2%) |  |
| **Insomnia (N)^d^** |  | | | |
| Never/rarely | 7963 (21.6%) | 82 (23.0%) | 7881 (21.8%) | 0.83 |
| Sometimes | 16831 (45.7%) | 163 (45.8%) | 16668 (46.1%) |  |
| Usually | 11756 (31.9%) | 111 (31.2%) | 11645 (32.2%) |  |
| **Insomnia diagnosis (N)** | 618 (1.7%) | 15 (4.1%) | 603 (1.7%) | <0.001 |
| **OSA diagnosis (N)** | 518 (1.4%) | 15 (4.1%) | 503 (1.4%) | <0.001 |
| **Snorers (N)** | 12278 (33.4%) | 126 (34.7%) | 12152 (33.3%) | 0.62 |
| **Night shift work (N) ^e^** |  | | | |
| Never/rarely | 1117 (52.5%) | 5 (45.5%) | 1112 (52.5%) | 0.90 |
| Sometimes | 574 (26.9%) | 4 (36.4%) | 570 (26.9%) |  |
| Usually | 169 (7.9%) | 1 (9.1%) | 168 (7.9%) |  |
| Always | 269 (12.6%) | 1 (9.1%) | 268 (12.7%) |  |

SBP: systolic blood pressure; DBP: diastolic blood pressure; WMH: white matter hyperintensity; OSA: obstructive sleep apnoea.

Data was available in a 98.5%, b 90.2%, c 99.1%, d 99.3%, e 5.8%, of data, respectively.

## Supplementary Table 4. Demographics of SVD burden groups (normalised WMH).

Continuous variables are shown as mean (SD) and categorical variables as count (%).

| **Variable** | **Total** | **Q1** | **Q2** | **Q3** | **Q4** | **P value** |
| --- | --- | --- | --- | --- | --- | --- |
| **N** | 36246 | 9061 | 9061 | 9063 | 9061 | <0.001 |
| **Age (years)** | 64.02 (7.73) | 58.57 (6.53) | 62.43 (6.90) | 65.79 (6.70) | 69.30 (6.35) | <0.001 |
| **Male (N)** | 17123 (47.2%) | 3754 (41.4%) | 4138 (45.7%) | 4373 (48.3%) | 4858 (53.6%) | <0.001 |
| **White (N)** | 33092 (91.3%) | 8291 (91.5%) | 8299 (91.6%) | 8283 (91.4%) | 8245 (91.0%) | <0.001 |
| **SBP (mmHg)** | 139.20 (18.57) | 132.39 (16.76) | 137.06 (17.86) | 141.16 (18.08) | 146.17 (18.71) | <0.001 |
| **DBP (mmHg)** | 77.98 (10.45) | 76.95 (10.09) | 77.66 (10.39) | 78.16 (10.49) | 79.15 (10.70) | <0.001 |
| **Waist-hip ratio** | 0.88 (0.09) | 0.85 (0.09) | 0.87 (0.09) | 0.88 (0.09) | 0.90 (0.09) | <0.001 |
| **History of ischaemic stroke (N)** | 352 (1.0%) | 35 (0.4%) | 52 (5.7%) | 115 (1.1%) | 181 (2.0%) | <0.001 |
| **Normalized WMH volume** | 0.01 (0.01) | 0.00 (0.00) | 0.00 (0.00) | 0.01 (0.00) | 0.02 (0.01) | <0.001 |
| **Sleep quality risk score^†^** | 2.47 (0.82) | 2.42 (0.81) | 2.47 (0.82) | 2.49 (0.82) | 2.49 (0.82) | <0.001 |
| **Sleep duration (N)^‡^** | | | | | | <0.001 |
| Normal | 24632 (68.5%) | 6300 (70.0%) | 6156 (68.4%) | 6128 (68.1%) | 6048 (67.6%) |  |
| Short | 8701 (24.2%) | 2212 (24.6%) | 2240 (24.9%) | 2184 (24.3%) | 2064 (23.1%) |  |
| Long | 2602 (7.2%) | 487 (5.4%) | 600 (6.7%) | 681 (7.8%) | 834 (9.3%) |  |
| **Insomnia (N)^§^** | | | | | | 0.49 |
| Never/rarely | 7832 (21.8%) | 2026 (22.5%) | 1944 (21.6%) | 1926 (24.3%) | 1936 (21.6%) |  |
| Sometimes | 16592 (46.1%) | 4153 (46.0%) | 4157 (46.2%) | 4184 (46.5%) | 4098 (45.7%) |  |
| Usually | 11576 (32.2%) | 2845 (31.5%) | 2903 (32.2%) | 2892 (32.1%) | 2936 (32.7%) |  |
| **Insomnia diagnosis (N)** | 616 (1.7%) | 97 (1.1%) | 140 (1.5%) | 183 (2.0%) | 196 (2.2%) | 0.002 |
| **OSA diagnosis (N)** | 516 (1.4%) | 80 (0.9%) | 114 (1.3%) | 149 (1.6%) | 173 (1.9%) | <0.001 |
| **Snorers (N)** | 12112 (33.4%) | 2878 (31.8%) | 3062 (33.8%) | 3151 (34.8%) | 3021 (33.3%) | <0.001 |
| **Night shift work (N)^¶^** | | | | | | 0.42 |
| Never/rarely | 1087 (52.1%) | 403 (50.5%) | 312 (51.9%) | 228 (53.1%) | 135 (53.8%) |  |
| Sometimes | 565 (27.1%) | 219 (27.4%) | 170 (28.3%) | 119 (27.7%) | 57 (22.7%) |  |
| Usually | 167 (8.0%) | 75 (9.4%) | 41 (6.8%) | 33 (7.7%) | 18 (7.2%) |  |
| Always | 269 (12.9%) | 101 (12.6%) | 78 (12.8%) | 49 (11.4%) | 41 (16.3%) |  |

SBP: systolic blood pressure; DBP: diastolic blood pressure; WMH: white matter hyperintensity; OSA: obstructive sleep apnoea.

Data was available in † 91.2%, ‡ 99.1%, § 99.6%, ¶ 8.8%, of data, respectively

## Supplementary Table 5. Tukey post-hoc analysis of NVC difference between time-of-day groups, adjusted for age and sex.

| **Time bins (hours)** | **(A) Time Group**  **(Time-of-day, hours)** | **(B) Time Group**  **(Time-of-day, hours)** | **(A-B) Mean Differences** | **Adjusted P-value** |
| --- | --- | --- | --- | --- |
| **3** | **8-11** | 11-14 | 0.076 | **<0.01** |
|  |  | 14-17 | -0.037 | 0.22 |
|  |  | 17-20 | -0.005 | 1.00 |
|  | **11-14** | 14-17 | -0.113 | **<0.001** |
|  |  | 17-20 | -0.077 | **<0.01** |
|  | **14-17** | 17-20 | 0.037 | 0.34 |
| **2** | **8-10** | 10-12 | 0.049 | 0.33 |
|  |  | 12-14 | -0.059 | 0.14 |
|  |  | 14-16 | -0.041 | 0.52 |
|  |  | 16-18 | -0.036 | 0.68 |
|  |  | 18-20 | 0.030 | 0.92 |
|  | **10-12** | 12-14 | 0.107 | 1.00 |
|  |  | 14-16 | -0.090 | **<0.01** |
|  |  | 16-18 | -0.085 | **<0.01** |
|  |  | 18-20 | -0.018 | 0.99 |
|  | **12-14** | 14-16 | -0.101 | **<0.001** |
|  |  | 16-18 | -0.095 | **<0.01** |
|  |  | 18-20 | -0.029 | 0.93 |
|  | **14-16** | 16-18 | 0.006 | 1.00 |
|  |  | 18-20 | 0.072 | 0.15 |
|  | **16-18** | 18-20 | 0.066 | 0.23 |

## Supplementary Table 6. Tukey post-hoc analysis of NVC difference between time-of-day groups (3-hour bins), stratified by age and sex.

| **Time bins (hours)** | **Group** | **Age Groups (years)** | **(A) Time Group (Time-of-day, hours)** | **(B) Time Group (Time-of-day, hours)** | **(A-B) Mean Differences** | **Adjusted P-value** |
| --- | --- | --- | --- | --- | --- | --- |
| **3** | **Female** | **< 60** | **8-11** | 11-14 | 0.152 | **<0.01** |
|  |  |  |  | 14-17 | -0.028 | 0.92 |
|  |  |  |  | 17-20 | 0.041 | 0.85 |
|  |  |  | **11-14** | 14-17 | -0.124 | **<0.05** |
|  |  |  |  | 17-20 | -0.111 | 0.13 |
|  |  |  | **14-17** | 17-20 | 0.013 | 0.99 |
|  |  | **60-69** | **8-11** | 11-14 | 0.011 | 0.99 |
|  |  |  |  | 14-17 | -0.063 | 0.43 |
|  |  |  |  | 17-20 | -0.065 | 0.55 |
|  |  |  | **11-14** | 14-17 | -0.073 | 0.22 |
|  |  |  |  | 17-20 | -0.076 | 0.36 |
|  |  |  | **14-17** | 17-20 | -0.002 | 1.00 |
|  |  | **≥ 70** | **8-11** | 11-14 | 0.079 | 0.54 |
|  |  |  |  | 14-17 | -0.049 | 0.85 |
|  |  |  |  | 17-20 | 0.048 | 0.91 |
|  |  |  | **11-14** | 14-17 | -0.128 | 0.06 |
|  |  |  |  | 17-20 | -0.031 | 0.96 |
|  |  |  | **14-17** | 17-20 | 0.096 | 0.47 |
|  | **Male** | **< 60** | **8-11** | 11-14 | 0.140 | **<0.05** |
|  |  |  |  | 14-17 | -0.018 | 0.98 |
|  |  |  |  | 17-20 | 0.007 | 1.00 |
|  |  |  | **11-14** | 14-17 | -0.158 | **<0.05** |
|  |  |  |  | 17-20 | -0.133 | 0.08 |
|  |  |  | **14-17** | 17-20 | 0.025 | 0.97 |
|  |  | **60-69** | **8-11** | 11-14 | 0.077 | 0.39 |
|  |  |  |  | 14-17 | -0.042 | 0.81 |
|  |  |  |  | 17-20 | -0.019 | 0.98 |
|  |  |  | **11-14** | 14-17 | -0.119 | 0.07 |
|  |  |  |  | 17-20 | -0.096 | 0.28 |
|  |  |  | **14-17** | 17-20 | 0.023 | 0.97 |
|  |  | **≥ 70** | **8-11** | 11-14 | 0.095 | 0.28 |
|  |  |  |  | 14-17 | -0.055 | 0.71 |
|  |  |  |  | 17-20 | 0.001 | 1.00 |
|  |  |  | **11-14** | 14-17 | -0.150 | **<0.05** |
|  |  |  |  | 17-20 | -0.094 | 0.36 |
|  |  |  | **14-17** | 17-20 | 0.056 | 0.75 |

## Supplementary Table 7. Tukey post-hoc analysis of NVC difference between time-of-day groups (2-hour bins), stratified by age and sex.

Female

| **Time bins (hours)** | **Group** | **Age Groups (years)** | **(A) Time Group**  **(Time-of-day, hours)** | **(B) Time Group**  **(Time-of-day, hours)** | **(A-B) Mean Differences** | **Adjusted P-value** |
| --- | --- | --- | --- | --- | --- | --- |
| **2** | **Female** | **< 60** | **8-10** | 10-12 | 0.091 | 0.51 |
|  |  |  |  | 12-14 | 0.149 | 0.08 |
|  |  |  |  | 14-16 | 0.024 | 1.00 |
|  |  |  |  | 16-18 | 0.043 | 0.97 |
|  |  |  |  | 18-20 | 0.053 | 0.97 |
|  |  |  | **10-12** | 12-14 | 0.058 | 0.89 |
|  |  |  |  | 14-16 | -0.067 | 0.83 |
|  |  |  |  | 16-18 | -0.048 | 0.95 |
|  |  |  |  | 18-20 | -0.037 | 0.99 |
|  |  |  | **12-14** | 14-16 | -0.125 | 0.25 |
|  |  |  |  | 16-18 | -0.106 | 0.43 |
|  |  |  |  | 18-20 | -0.095 | 0.73 |
|  |  |  | **14-16** | 16-18 | 0.019 | 1.00 |
|  |  |  |  | 18-20 | 0.029 | 1.00 |
|  |  |  | **16-18** | 18-20 | 0.011 | 1.00 |
|  |  | **60-69** | **8-10** | 10-12 | -0.026 | 1.00 |
|  |  |  |  | 12-14 | 0.014 | 1.00 |
|  |  |  |  | 14-16 | -0.044 | 0.96 |
|  |  |  |  | 16-18 | -0.130 | 0.15 |
|  |  |  |  | 18-20 | -0.014 | 1.00 |
|  |  |  | **10-12** | 12-14 | 0.040 | 0.96 |
|  |  |  |  | 14-16 | -0.018 | 1.00 |
|  |  |  |  | 16-18 | -0.103 | 0.30 |
|  |  |  |  | 18-20 | 0.012 | 1.00 |
|  |  |  | **12-14** | 14-16 | -0.058 | 0.81 |
|  |  |  |  | 16-18 | -0.144 | **<0.05** |
|  |  |  |  | 18-20 | 0.029 | 1.00 |
|  |  |  | **14-16** | 16-18 | 0.085 | 0.48 |
|  |  |  |  | 18-20 | 0.030 | 1.00 |
|  |  |  | **16-18** | 18-20 | 0.115 | 0.46 |
|  |  | **≥ 70** | **8-10** | 10-12 | -0.056 | 0.98 |
|  |  |  |  | 12-14 | 0.017 | 1.00 |
|  |  |  |  | 14-16 | -0.106 | 0.73 |
|  |  |  |  | 16-18 | -0.097 | 0.85 |
|  |  |  |  | 18-20 | 0.042 | 1.00 |
|  |  |  | **10-12** | 12-14 | 0.073 | 0.88 |
|  |  |  |  | 14-16 | -0.052 | 0.97 |
|  |  |  |  | 16-18 | -0.041 | 0.99 |
|  |  |  |  | 18-20 | 0.098 | 0.89 |
|  |  |  | **12-14** | 14-16 | -0.125 | 0.31 |
|  |  |  |  | 16-18 | -0.114 | 0.53 |
|  |  |  |  | 18-20 | 0.025 | 1.00 |
|  |  |  | **14-16** | 16-18 | 0.011 | 1.00 |
|  |  |  |  | 18-20 | 0.150 | 0.54 |
|  |  |  | **16-18** | 18-20 | 0.139 | 0.67 |

Male

| **Time bins (hours)** | **Group** | **Age Groups (years)** | **(A) Time Group**  **(Time-of-day, hours)** | **(B) Time Group**  **(Time-of-day, hours)** | **(A-B) Mean Differences** | **Adjusted P-value** |
| --- | --- | --- | --- | --- | --- | --- |
| **2** | **Male** | **< 60** | **8-10** | 10-12 | 0.100 | 0.43 |
|  |  |  |  | 12-14 | 0.146 | 0.15 |
|  |  |  |  | 14-16 | -0.021 | 1.00 |
|  |  |  |  | 16-18 | 0.005 | 1.00 |
|  |  |  |  | 18-20 | 0.036 | 0.99 |
|  |  |  | **10-12** | 12-14 | 0.046 | 0.98 |
|  |  |  |  | 14-16 | -0.121 | 0.42 |
|  |  |  |  | 16-18 | -0.096 | 0.59 |
|  |  |  |  | 18-20 | -0.064 | 0.95 |
|  |  |  | **12-14** | 14-16 | -0.167 | 0.16 |
|  |  |  |  | 16-18 | -0.141 | 0.71 |
|  |  |  |  | 18-20 | -0.110 | 1.00 |
|  |  |  | **14-16** | 16-18 | 0.026 | 0.97 |
|  |  |  |  | 18-20 | 0.057 | 1.00 |
|  |  |  | **16-18** | 18-20 | 0.032 | 1.00 |
|  |  | **60-69** | **8-10** | 10-12 | 0.078 | 0.75 |
|  |  |  |  | 12-14 | 0.038 | 0.99 |
|  |  |  |  | 14-16 | -0.091 | 0.63 |
|  |  |  |  | 16-18 | 0.003 | 1.00 |
|  |  |  |  | 18-20 | 0.011 | 1.00 |
|  |  |  | **10-12** | 12-14 | -0.040 | 0.99 |
|  |  |  |  | 14-16 | -0.170 | **<0.05** |
|  |  |  |  | 16-18 | -0.075 | 0.78 |
|  |  |  |  | 18-20 | -0.067 | 0.93 |
|  |  |  | **12-14** | 14-16 | -0.129 | 0.29 |
|  |  |  |  | 16-18 | -0.035 | 0.99 |
|  |  |  |  | 18-20 | -0.027 | 1.00 |
|  |  |  | **14-16** | 16-18 | 0.095 | 0.59 |
|  |  |  |  | 18-20 | 0.103 | 0.71 |
|  |  |  | **16-18** | 18-20 | 0.008 | 1.00 |
|  |  | **≥ 70** | **8-10** | 10-12 | 0.143 | 0.26 |
|  |  |  |  | 12-14 | 0.076 | 0.86 |
|  |  |  |  | 14-16 | 0.000 | 1.00 |
|  |  |  |  | 16-18 | -0.050 | 0.97 |
|  |  |  |  | 18-20 | 0.068 | 0.96 |
|  |  |  | **10-12** | 12-14 | -0.068 | 0.88 |
|  |  |  |  | 14-16 | -0.143 | 0.17 |
|  |  |  |  | 16-18 | -0.193 | **<0.05** |
|  |  |  |  | 18-20 | -0.075 | 0.80 |
|  |  |  | **12-14** | 14-16 | -0.076 | 0.30 |
|  |  |  |  | 16-18 | -0.126 | 1.00 |
|  |  |  |  | 18-20 | -0.008 | 0.96 |
|  |  |  | **14-16** | 16-18 | -0.050 | 0.95 |
|  |  |  |  | 18-20 | 0.068 | 0.66 |
|  |  |  | **16-18** | 18-20 | 0.118 | 0.65 |

## Supplementary Table 8. Cosinor analysis.

| **Model** | **Groups** | **Parameters** | **Cosinor with 12-h period** | **Cosinor with 24-h period** |
| --- | --- | --- | --- | --- |
| **Sex (age adjusted)** | **Female** | **Amplitude** | 0.05 (0.02 to 0.08) *** | 0.06 (-0.01 to 0.14) |
|  |  | **Acrophase** | 3.08 (2.48 to 3.67) *** | 0.13 (-0.43 to 0.68) |
|  | **Male** | **Amplitude** | 0.07 (0.04 to 0.10) *** | 0.12 (0.04 to 0.19) ** |
|  |  | **Acrophase** | 3.05 (2.61 to 3.49) *** | 0.22 (-0.06 to 0.50) |
| **Age (sex adjusted)** | **< 60 years** | **Amplitude** | 0.07 (0.03 to 0.11) *** | 0.15 (-0.06 to 0.25) ** |
|  |  | **Acrophase** | -2.75 (-3.25 to -2.25) *** | 0.41 (0.17 to 0.65) *** |
|  | **60-69 years** | **Amplitude** | 0.05 (0.02 to 0.08) *** | 0.06 (0.14 to 0.09) |
|  |  | **Acrophase** | 2.83 (2.22 to 3.45) *** | -0.06 (-0.79 to 0.67) |
|  | **≥ 70 years** | **Amplitude** | 0.07 (0.03 to 0.10) *** | 0.05 (-0.03 to 0.14) |
|  |  | **Acrophase** | 2.76 (2.13 to 3.39) *** | -0.24 (-1.58 to 1.11) |

## Supplementary Table 9. Cosinor analysis of stroke.

| **Model** | **Ischaemic stroke history** | **Parameters** | **Cosinor with 12-h period** | **Cosinor with 24-h period** |
| --- | --- | --- | --- | --- |
| **Unadjusted** | **Yes** | **Amplitude** | 0.07 (-0.15 to 0.28) | 0.19 (-0.29 to 0.68) |
|  |  | **Acrophase** | 0.22 (-2.69 to 3.13) | 3.09 (1.47 to 4.72) *** |
|  | **No** | **Amplitude** | 0.05 (0.03 to 0.07) *** | 0.07 (0.02 to 0.12) ** |
|  |  | **Acrophase** | 2.89 (2.50 to 3.28) *** | 0.06 (-0.33 to 0.46) |
| **Age and sex-adjusted** | **Yes** | **Amplitude** | 0.07 (-0.14 to 0.28) | 0.21 (-0.28 to 0.70) |
|  |  | **Acrophase** | 0.27 (-2.49 to 3.03) | 3.34 (1.70 to 4.58) *** |
|  | **No** | **Amplitude** | 0.06 (0.04 to 0.08) *** | 0.08 (0.03 to 0.13) ** |
|  |  | **Acrophase** | 2.95 (2.59 to 3.31) *** | 0.09 (-0.23 to 0.41) |
| **Age, sex and vascular risk factors-adjusted** | **Yes** | **Amplitude** | 0.04 (-0.18 to 0.26) | 0.16 (-0.37 to 0.68) |
|  |  | **Acrophase** | 0.96 (-4.64 to 6.56) | -3.01 (-4.69 to -1.33) *** |
|  | **No** | **Amplitude** | 0.06 (0.04 to 0.08) *** | 0.08 (0.03 to 0.14) ** |
|  |  | **Acrophase** | 2.97 (2.61 to 3.33) *** | 0.10 (-0.21 to 0.41) |

## Supplementary Table 10. Cosinor analysis of cerebral small vessel disease burden.

| **Model** | **Quartiles** | **Parameters** | **Cosinor with 12-h period** | **Cosinor with 24-h period** |
| --- | --- | --- | --- | --- |
| **Unadjusted** | **Q1** | **Amplitude** | 0.05 (0.01 to 0.09) *** | 0.04 (-0.03 to 0.12) |
|  |  | **Acrophase** | 2.57 (1.71 to 3.52) *** | -0.52 (-2.54 to 1.50) |
|  | **Q2** | **Amplitude** | 0.05 (0.01 to 0.09) * | 0.10 (0.00 to 0.12) |
|  |  | **Acrophase** | -2.87 (-3.69 to -2.05) *** | 0.38 (-0.03 to 0.79) |
|  | **Q3** | **Amplitude** | 0.09 (0.05 to 0.13) *** | 0.07 (0.02 to 0.12) ** |
|  |  | **Acrophase** | 2.46 (1.96 to 2.95) *** | -0.91 (-2.52 to 0.71) |
|  | **Q4** | **Amplitude** | 0.05 (0.01 to 0.10) * | 0.02 (-0.03 to 0.07) |
|  |  | **Acrophase** | 2.45 (1.55 to 3.34) *** | -1.30 (-6.64 to 4.04) |
| **Age and sex-adjusted** | **Q1** | **Amplitude** | 0.06 (0.02 to 0.10) ** | 0.07 (-0.02 to 0.18) |
|  |  | **Acrophase** | 2.82 (2.11 to 3.52) *** | -0.11 (-0.94 to 0.72) |
|  | **Q2** | **Amplitude** | 0.06 (0.01 to 0.10) ** | 0.13 (0.03 to 0.24) * |
|  |  | **Acrophase** | -2.72 (-3.39 to -2.04) *** | 0.42 (0.10 to 0.74) ** |
|  | **Q3** | **Amplitude** | 0.08 (0.04 to 0.12) *** | 0.08 (0.00 to 0.16) * |
|  |  | **Acrophase** | 2.65 (2.13 to 3.16) *** | -0.43 (-1.53 to 0.67) |
|  | **Q4** | **Amplitude** | 0.05 (0.01 to 0.09) ** | 0.03 (-0.06 to 0.12) |
|  |  | **Acrophase** | 2.65 (1.82 to 3.47) *** | -0.32 (-3.18 to 2.53) |
| **Age, sex and vascular risk factors-adjusted** | **Q1** | **Amplitude** | 0.06 (0.02 to 0.10) ** | 0.07 (-0.02 to 0.17) |
|  |  | **Acrophase** | 2.80 (2.12 to 3.48) *** | -0.12 (-0.94 to 0.69) |
|  | **Q2** | **Amplitude** | 0.06 (0.02 to 0.10) ** | 0.14 (0.04 to 0.25) *** |
|  |  | **Acrophase** | -2.67 (-3.32 to -2.03) *** | 0.42 (0.12 to 0.72) *** |
|  | **Q3** | **Amplitude** | 0.08 (0.04 to 0.12) *** | 0.08 (0.00 to 0.16) |
|  |  | **Acrophase** | 2.66 (2.13 to 3.18) *** | -0.41(-1.49 to 0.67) |
|  | **Q4** | **Amplitude** | 0.05 (0.01 to 0.09) * | 0.02 (-0.06 to 0.11) |
|  |  | **Acrophase** | 2.63 (1.70 to 3.56) *** | -0.41(-4.31 to 3.49) |

## Supplementary Table 11. Tukey post-hoc analysis of NVC difference between time-of-day groups, stratified by sleep duration, adjusted for age, sex and vascular risk factors.

| **Time bins (hours)** | **(A) Time Group**  **(Time-of-day, hours)** | **(B) Time Group**  **(Time-of-day, hours)** | **(A-B) Mean Differences** | **Adjusted P-value** |
| --- | --- | --- | --- | --- |
| **3** | **8-11** | 11-14 | 0.076 | **<0.01** |
|  |  | 14-17 | -0.037 | 0.22 |
|  |  | 17-20 | -0.005 | 1.00 |
|  | **11-14** | 14-17 | -0.113 | **<0.001** |
|  |  | 17-20 | -0.077 | **<0.01** |
|  | **14-17** | 17-20 | 0.037 | 0.34 |
| **2** | **8-10** | 10-12 | 0.049 | 0.33 |
|  |  | 12-14 | -0.059 | 0.14 |
|  |  | 14-16 | -0.041 | 0.52 |
|  |  | 16-18 | -0.036 | 0.68 |
|  |  | 18-20 | 0.030 | 0.92 |
|  | **10-12** | 12-14 | 0.107 | 1.00 |
|  |  | 14-16 | -0.090 | **<0.01** |
|  |  | 16-18 | -0.085 | **<0.01** |
|  |  | 18-20 | -0.018 | 0.99 |
|  | **12-14** | 14-16 | -0.101 | **<0.001** |
|  |  | 16-18 | -0.095 | **<0.01** |
|  |  | 18-20 | -0.029 | 0.93 |
|  | **14-16** | 16-18 | 0.006 | 1.00 |
|  |  | 18-20 | 0.072 | 0.15 |
|  | **16-18** | 18-20 | 0.066 | 0.23 |

## Reference

1. Miller KL, Alfaro-Almagro F, Bangerter NK, et al. Multimodal population brain imaging in the UK Biobank prospective epidemiological study. *Nat Neurosci*. Nov 2016;19(11):1523-1536. doi:10.1038/nn.4393

2. Alfaro-Almagro F, Jenkinson M, Bangerter NK, et al. Image processing and Quality Control for the first 10,000 brain imaging datasets from UK Biobank. *Neuroimage*. Feb 1 2018;166:400-424. doi:10.1016/j.neuroimage.2017.10.034

3. M. Jenkinson MP, S. Smith. BET2: MR-based estimation of brain, skull and scalp surfaces. presented at: Eleventh Annual Meeting of the Organization for Human Brain Mapping; June 12-16 2005; Toronto, Ontario, Canada.

4. Zhang Y, Brady M, Smith S. Segmentation of brain MR images through a hidden Markov random field model and the expectation-maximization algorithm. *IEEE Trans Med Imaging*. Jan 2001;20(1):45-57. doi:10.1109/42.906424

5. Wardlaw JM, Smith EE, Biessels GJ, et al. Neuroimaging standards for research into small vessel disease and its contribution to ageing and neurodegeneration. *Lancet Neurol*. Aug 2013;12(8):822-38. doi:10.1016/S1474-4422(13)70124-8

6. Griffanti L, Zamboni G, Khan A, et al. BIANCA (Brain Intensity AbNormality Classification Algorithm): A new tool for automated segmentation of white matter hyperintensities. *Neuroimage*. Nov 1 2016;141:191-205. doi:10.1016/j.neuroimage.2016.07.018

7. Fan M, Sun D, Zhou T, et al. Sleep patterns, genetic susceptibility, and incident cardiovascular disease: a prospective study of 385 292 UK biobank participants. *Eur Heart J*. Mar 14 2020;41(11):1182-1189. doi:10.1093/eurheartj/ehz849
